# LncRNA‐encoded microproteins: A new form of cargo in cell culture‐derived and circulating extracellular vesicles

**DOI:** 10.1002/jev2.12123

**Published:** 2021-07-12

**Authors:** Tanxi Cai, Qing Zhang, Bowen Wu, Jifeng Wang, Na Li, Tingting Zhang, Zhipeng Wang, Jianjun Luo, Xiaojing Guo, Xiang Ding, Zhensheng Xie, Lili Niu, Weihai Ning, Zhen Fan, Xiaowei Chen, Xiangqian Guo, Runsheng Chen, Hongwei Zhang, Fuquan Yang

**Affiliations:** ^1^ Laboratory of Protein and Peptide Pharmaceuticals and Laboratory of Proteomics Institute of Biophysics Chinese Academy of Sciences Beijing China; ^2^ University of Chinese Academy of Sciences Beijing China; ^3^ Key Laboratory of RNA Biology Institute of Biophysics Chinese Academy of Sciences Beijing China; ^4^ Department of Neurosurgery Sanbo Brain Hospital Capital Medical University Beijing China; ^5^ Center for High Throughput Sequencing Core Facility for Protein Research Institute of Biophysics Chinese Academy of Sciences Beijing China; ^6^ Henan Provincial Engineering Centre for Tumour Molecular Medicine School of Basic Medical Sciences Henan University Kaifeng China

**Keywords:** cancer, extracellular vesicles (EVs), lncRNA‐encoded microproteins, long noncoding RNA (lncRNA), small open reading frames (smORFs)

## Abstract

Advancements in omics‐based technologies over the past few years have led to the discovery of numerous biologically relevant peptides encoded by small open reading frames (smORFs) embedded in long noncoding RNA (lncRNA) transcripts (referred to as microproteins here) in a variety of species. However, the mechanisms and modes of action that underlie the roles of microproteins have yet to be fully characterized. Herein, we provide the first experimental evidence of abundant microproteins in extracellular vesicles (EVs) derived from glioma cancer cells, indicating that the EV‐mediated transfer of microproteins may represent a novel mechanism for intercellular communication. Intriguingly, when examining human plasma, 48, 11 and 3 microproteins were identified from purified EVs, whole plasma and EV‐free plasma, respectively, suggesting that circulating microproteins are primarily enriched in EVs. Most importantly, the preliminary data showed that the expression profile of EV microproteins in glioma patient diverged from the health donors, suggesting that the circulating microproteins in EVs might have potential diagnostic application in identifying patients with glioma.

## 1 INTRODUCTION

Long noncoding RNAs (lncRNAs) are a family of non‐coding RNAs (ncRNAs) exceeding 200 nucleotides in length that were initially considered as ‘junk RNAs’, as they possess no apparent open reading frames (ORFs) for proteins > 100 amino acids (aa). However, advancements in bioinformatics, transcriptomics and proteomics technologies over the past few years have led to the discovery of numerous new peptides encoded by small open reading frames (smORFs) embedded in lncRNA transcripts (referred as microproteins here) (Hanyu‐Nakamura et al., 2008; Matsumoto et al., 2017; Nelson et al., 2016). Accumulated evidence indicates that these microproteins are involved in diverse regulatory roles in many vital processes, such as Ca^2+^ homeostasis (Jackson et al., 2018), metabolism (Huang et al., 2017), development (Makarewich et al., 2018), stress signalling (Pircher et al., 2014) and DNA repair (Slavoff et al., 2014). Furthermore, numerous cancer‐related lncRNAs and its encoded functional microproteins were found to be able to influence tumorigenesis, invasion and metastasis. Jiang et al. reported that decreased LINC00961 was associated with advanced clinical stage, lymph node metastasis, and shorter survival time of non‐small‐cell lung carcinoma patients (Jiang et al., 2018). Matsumoto et al. further demonstrated that LINC00961 is tranlatable. Its encoded 90‐aa‐long polypeptide, termed ‘small regulatory polypeptide of amino acid response (SPAR)’, inhibits amino acid‐mediated mTORC1 activation at the lysosomal membrane (Matsumoto et al., 2017). The NOBODY peptide—a 71‐aa‐long peptide encoded by the lncRNA LINC01420—was demonstrated to interact with a mRNA capping protein, removing the 5′ cap from mRNAs to promote 5′‐3′ decay (D'Lima et al., 2017). The level of Nobody is anticorrelated with cellular P‐body numbers, which regulate the homeostasis of endogenous cellular nonsense‐mediate decay substrates. Even though the effects of this process on tumour growth, development, and metabolism are unclear, LINC01420 knockdown significantly inhibits nasopharyngeal carcinoma (NPC) cell invasion (Yang et al., 2017). Huang et al. reported that the function of a 53‐aa‐long conserved peptide encoded by HOXB‐AS3 appeared to be downregulated in highly metastatic colon, breast, nasopharyngeal and ovarian cancer cells, as well as primary colorectal cancer (CRC) tissues (Huang et al., 2017). The functional diversity of these microproteins, as well as their roles in cancer development, has attracted the attention of the scientific community (Choi et al., 2019; Matsumoto & Matsumoto, 2018; Rion & Rüegg, 2017; Yeasmin et al., 2018).

However, although the limited numbers of microproteins have been functionally characterized, the mechanisms that underlie their roles in cancers remain uncharacterized. Many issues, such as possible modes of action, have yet to be explored. Of particular interest is whether microproteins are taking part in intercellular communication between tumour cells and stromal cells in either the local or distant micro‐environment. Considering that neoplastic cells use various intercellular communication mechanisms to adapt to the local microenvironment, manipulate the immune system, and facilitate metastasis, this is of great interest (Hanahan & Weinberg, 2011). During the past few years, intense and exciting research in extracellular vesicles (EVs) has indicated that EVs represent a novel mode of intercellular communication. EVs deliver a variety of biomolecules including proteins, lipids, DNA, mRNA, microRNAs (miRNAs) and lncRNAs to recipient cells, mediating cell activation, phenotypic modification, and the reprogramming of cellular function (Maas et al., 2017; Quesenberry et al., 2014). Notably, numerous lncRNAs have been found to be selectively sorted into EVs, regulating cancer onset and progression in a variety of ways, making lncRNAs an intensely researched topic for cancer prediction (Huarte, 2015). It is therefore conceivable that EVs and microproteins might also function together to disseminate molecules and signals for the purpose of changing or regulating recipient cells locally or at distance. And yet, to our knowledge, whether there are microproteins, especially functional microproteins in the cell culture‐derived or circulating EVs remains unknown.

In the present study, we employed multiple technologies—including an improved microprotein analytical workflow and stringent criteria—to provide the first experimental evidence of abundant microproteins in cellular and circulating EVs, suggesting EV‐mediated transfer of microproteins as a novel mechanism for intercellular communication. Furthermore, our preliminary data showed that microproteins were enriched in circulating EVs and could be used to distinguish glioma cancer patients from healthy donors.

## 2 RESULTS

### 2.1 Identification of microproteins from glioma cancer cells and cell culture‐derived EVs

**FIGURE 1:**
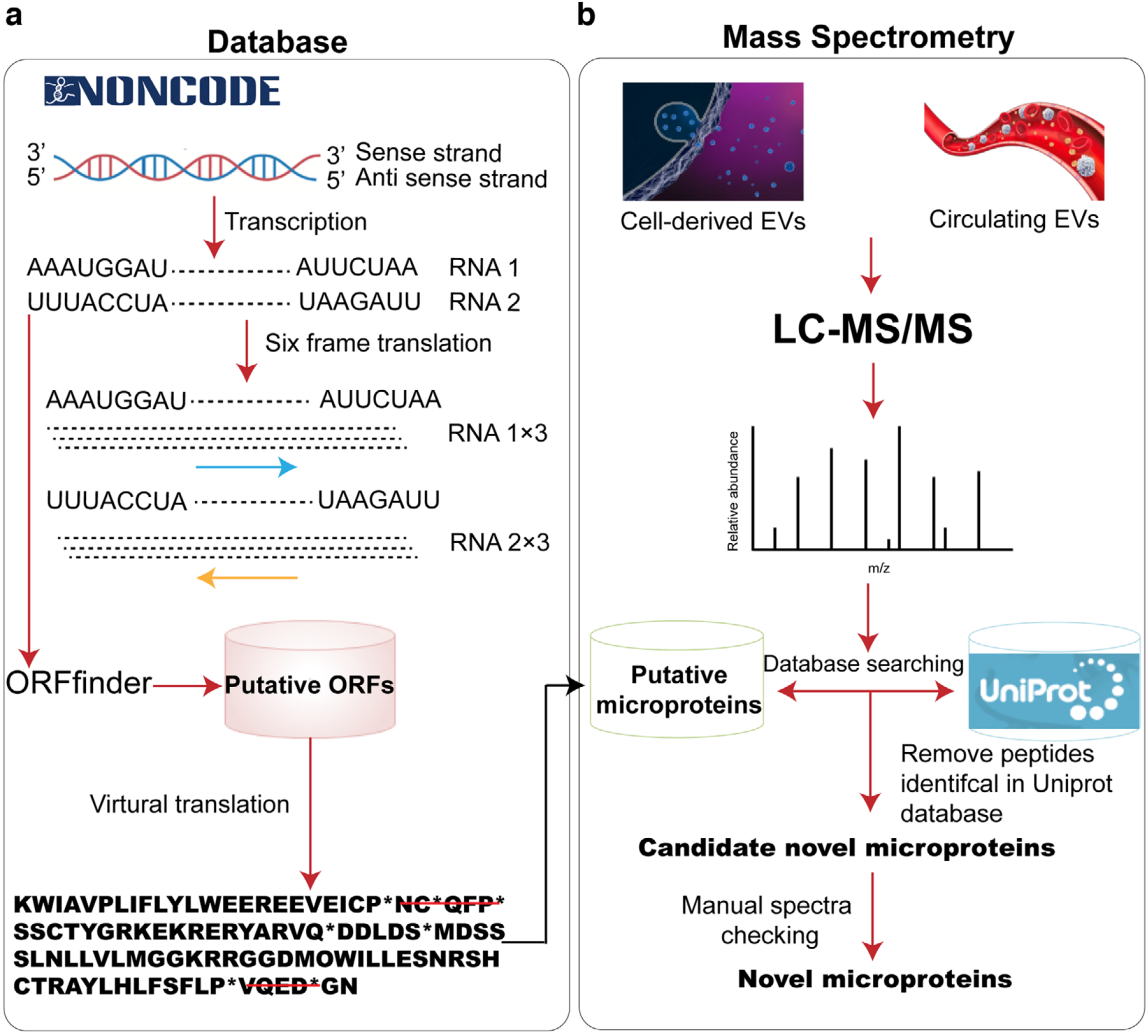
Schematic illustration of the workflow for MS‐based discovery of microproteins encoded by lncRNAs. (a) Construction of the putative microprotein database. The human lncRNA transcripts deposited in noncode database were screened by oRFfinder and six‐frame translation to find all possible smORFs and then theoretically translated into putative microproteins. All microproteins were collected into the human microprotein database. (b) MS‐based identification of microproteins in cell culture‐derived and circulating EVs

It is challenging to achieve high sensitivity and specificity in computational sequencing analysis for the prediction of smORFs embedded in lncRNA transcripts, and deep sequencing‐based ribosome profiling only provides indirect evidence of translation. As a result, MS‐based proteomics—directly detecting the peptides generated from smORFs—has become a powerful tool for identifying microproteins from different biological samples (Ma et al., 2016; Matsumoto & Nakayama, 2018). However, MS‐based microprotein detection is still analytically challenging, as the number of microproteins identified by MS from different biological samples is relatively small (Fouzia et al., 2018). In order to improve the discovery of novel microproteins from cancer cells and cell culture‐derived EVs, we developed a fine‐tuned MS‐based microprotein analytical workflow (Figure 1). Since tandem MS‐based database searching is the crucial step for MS‐based identification of microproteins, we developed a microprotein database that could entirely cover the putative microproteins in humans but avoid generating a dataset as excessively large as the six‐frame translation of the entire genome. To this end, the human lncRNA transcripts deposited in the NONCODE (http://www.noncode.org/) database, which represents an interactive database containing the most complete collection and annotation of lncRNAs, were scanned by ORFfinder and six‐frame translation mode to obtain all possible smORFs, and then theoretically translated into microproteins. In comparison to smaller databases utilising RNA transcripts from RNA‐seq or Ribo‐seq—which only capture actively translated RNA transcripts and rely greatly on the depth of the sequencing—the in‐house microprotein database is a medium size, containing 3,969,981 putative microproteins. To verify the quality of the database, recently reported functional microproteins such as Myoregulin (Anderson et al., 2015), Myomixer (Bi et al., 2017), Minion (Zhang et al., 2017), HOXB‐AS3 (Huang et al., 2017), SPAR (Matsumoto et al., 2017), NoBody (Pircher et al., 2014) and LINC‐PINT (Zhang et al., 2018) were selected and blasted within the database. All of these microproteins were found in our database, validating its high quality and comprehensiveness.

**FIGURE 2:**
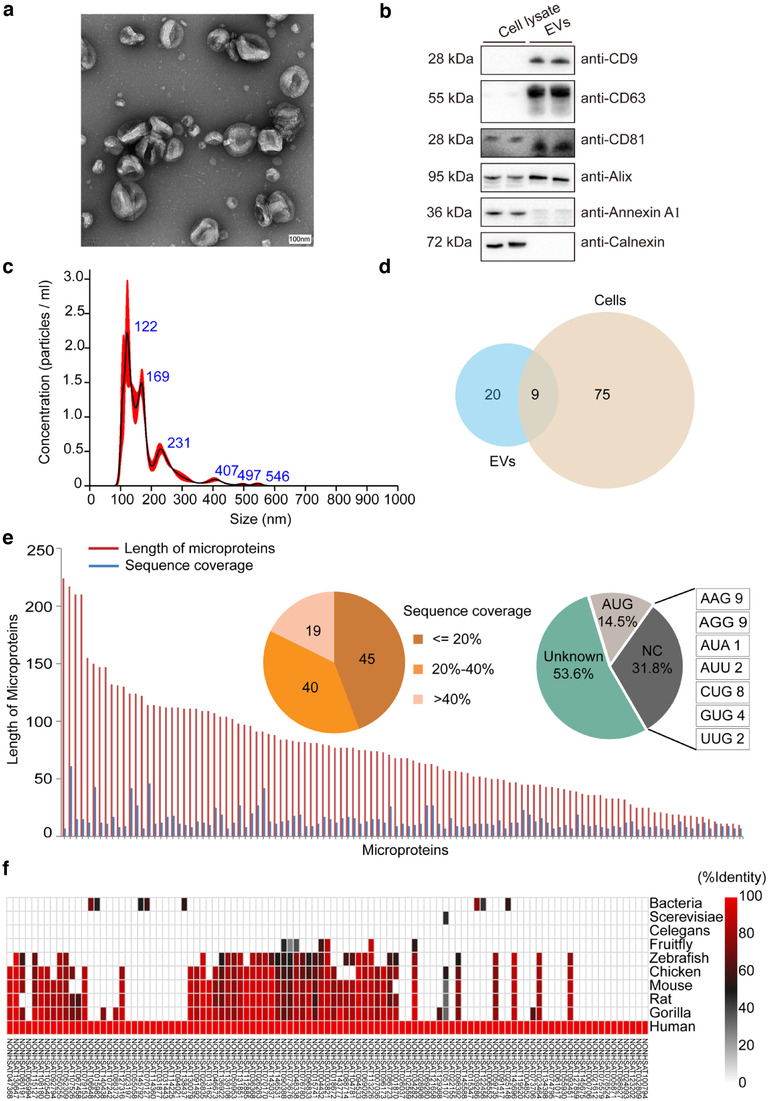
Identification of microproteins from glioma cancer cells and cell culture‐derived EVs. (a) TEM image of glioma cell culture‐derived EVs. (b) Western blot of commonly used EV marker proteins, including CD9, CD63, CD81 and Alix, with a 15‐µg protein loading volume. (c) Nanoparticle tracking analysis (NTA) of EVs released from glioma cells. (d) Microproteins identified from glioma cells and cell culture‐derived EVs, respectively. (e) Length distribution, sequence coverage and start codon usage of new microproteins. The values in the sequence coverage pie chart refer to the number of individual microproteins identified. (f) Evolutionary conservatism of microproteins. A sequence similarity search between the amino acid sequences of identified microproteins with that from other species was performed with the blast (basic local alignment search tool) program in uniprot database (https://www.uniprot.org/). The colour scale indicates the sequence identity

To identify novel microproteins, we applied the improved workflow to data mining on a well‐established dataset (collected previously in our lab) of Tandem Mass Tag (TMT)‐based quantitative analytics on glioma cancer cells and cell culture‐derived EVs. Purified EVs of glioma cancer cells were examined by transmission electron microscopy (TEM). TEM imaging showed that the purified EVs have a typical cup‐shaped structure, which is an artefact of the fixation process (Figure 2a). Western Blot experiments using antibodies to commonly used EV marker proteins, such as CD9, CD63, CD81 and Alix, were also carried out. The results demonstrated that CD9, CD63, CD81 and Alix were all enriched in isolated EVs when compared to cell lysates (Figure 2b). Meanwhile, the almost undetectable signals of Calnexin (a marker of endoplasmic reticulum) in the EV fraction further reflect the high purity of purified EVs, while the low abundance of Annexin A1 (a marker of microvesicles) detected in the EV fraction suggests an enrichment for exosomes. Particle concentrations and size distributions were determined with nanoparticle tracking analysis (NTA). The size distribution was showed in the Figure 2c, and the concentration was about 3.48 × 10^8^ particles/per million cells.

Using a series of stringent criteria, 84 and 29 microproteins were confidently identified from glioma cells and EVs, respectively, with nine microproteins identified in both cells and EVs (Figure 2d). Representative mass spectra of several peptides of identified microproteins are listed in the Figure S1. Meanwhile, a fraction of microproteins were validated by comparing their MS^2^ spectra with synthetic peptide standards. Our results showed that strong matching between the MS‐identified peptides of the selected microproteins and their corresponding synthetic peptides were observed and representative spectra matching were shown in Figure S2.

Bioinformatics analysis revealed that most identified microproteins were found to have a length less than 100 aa, with the shortest at 20 aa long (Figure 2e). The length of a microprotein with a known start codon was determined by the predicted smORF length. For microproteins with an unknown start codon, the length was defined as the length of the interval between two nearby stop codons. The matched unique peptides validated the presence of novel microproteins, providing an average of 25.6% sequence coverage (Figure 2e). This number could further increase if supportive peptides—the non‐unique peptides that are assigned by the database search engine to certain canonical protein identification—were taken into consideration. Moreover, a potential explanation for those microproteins with lower sequence coverage (< 20%) could be their generally low expression levels in different tissues (< 10 RPKM) (Figure S3). Intriguingly, less than 15% of 104 identified human microproteins initiated with AUG. 32% started with near cognate codon, while most microproteins (53.6%) had an unknown start codon (Figure 2e).

To further determine the evolutionary conservation of microproteins—which is an important criterion of the coding propensity predicting algorithms—we performed a homology search of these microproteins against various genomes ranging from all genome‐sequenced bacteria to primates. The results showed that more than half of the microproteins identified in primates also exist in rodents, while less than 10% could be found in bacteria (Figure 2f). This indicates that these proteins are mostly from evolutionarily very young genes, which partially explains why the predicting algorithms have considered these genes as non‐coding. All of the identification information of the new microproteins, including lncRNA names, unique peptide sequences, peptide length and search engines can be found in Tables S1 and S2.

### 2.2 Experimental validation for the presence of microproteins in cells and cell culture‐derived EVs

**FIGURE 3:**
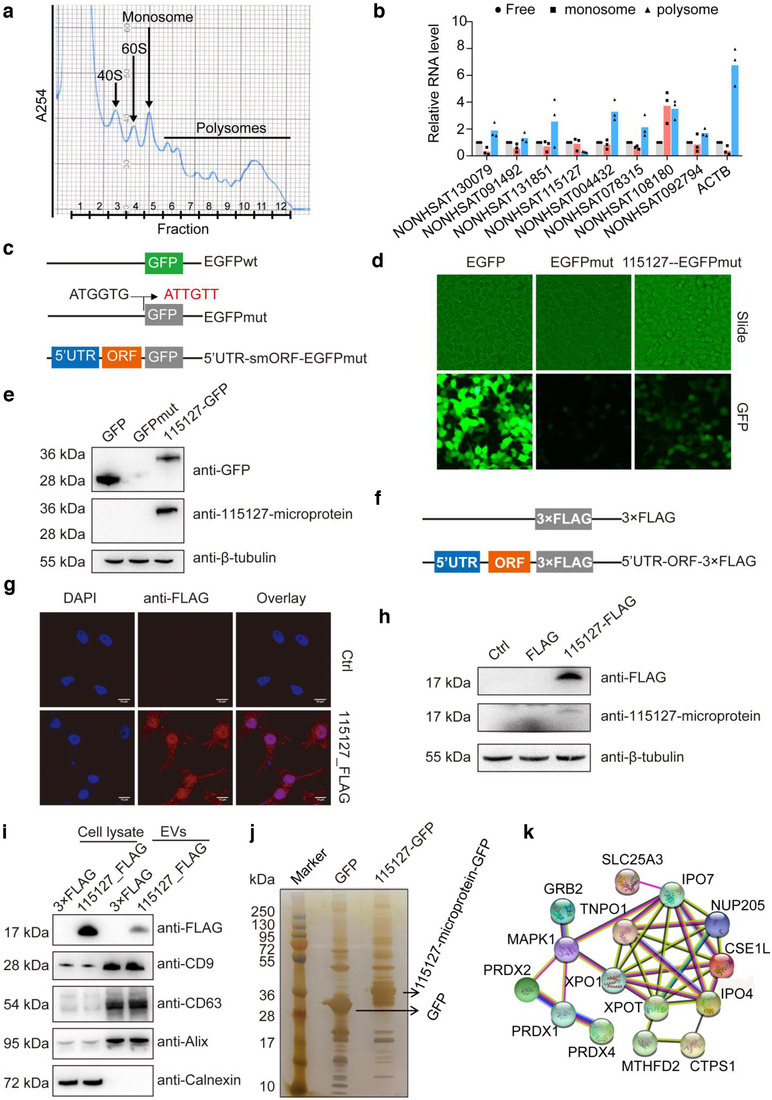
Experimental validation for the presence of microproteins in cells and cell culture‐derived EVs. (a) Polysome fraction of glioma cell lysate. golima cells were treated with 0.1 mg/ml cycloheximide, lysed and separated by sucrose gradient centrifugation. All fractions were collected. Fraction 1–4 were combined together and marked as R1, fraction 5 was marked as R2, and fraction 6–12 were combined together and marked as R3. total RNA from R1, R2 and R3 were isolated and marked as unbound (free) RNAs, monosome‐and polysome‐ bound RNAs, respectively. (b) Relative ratios of monosome‐ and polysome‐bound RNAs vs free RNAs for several microprotein‐encoded lncRNAs. (c) Diagram of the gfp fusion constructs used for transfection. The start codon ATGGTG of the gfp (GFPWT) gene is mutated to ATTGTT (GFPmut). (d) Expression of the NONHSAT115127‐gfp fusion protein in NONHSAT115127 5′ UTR‐smORF‐GFPmut‐transfected 293T cells. (e) Western blot analysis of cell lysates from 293T cells transfected with different gfp fusion constructs using anti‐gfp antibodies, with a 15‐µg protein loading volume. β‐tubulin was used as a protein loading control. (f) Diagram of the flag fusion construct used for transfection. (g) Expression of the NONHSAT115127‐flag fusion proteins in NONHSAT115127 5′ UTR‐smORF‐flag‐transfected cells. (h) Western blot analysis of cell lysates from 293T cells transfected with different flag fusion constructs using anti‐flag antibodies, with a 15‐µg protein loading volume. β‐tubulin was used as a protein loading control. (j) SDS‐PAGE of proteins co‐immunoprecipitated from NONHSAT115127 5′ UTR‐smORF‐GFP‐transfected cells via anti‐gfp antibodies. (k) Protein‐protein interaction networks. the protein‐protein interaction networks were analyzed based on the proteins that were detected in all three replicates and significantly upregulated (fold change ≧4.0, *p* < 0.05) in the co‐ip fraction derived from the NONHSAT115127 5 ’UTR‐smORF‐GFP‐transfected cells compared to the GFP‐transfected cells

To determine whether microprotein‐encoding lncRNAs are actively expressed, reverse transcription polymerase chain reaction (RT‐PCR) was employed to evaluate the expression of several microprotein‐encoded lncRNAs in glioma cells. The results showed that most of the microprotein‐encoding lncRNAs selected were actively expressed in glioma cells (Figure S4). Ribosomes were further purified from glioma cells and the monosome‐ and polysome‐associated lncRNAs were examined using quantitative RT‐PCR. The results showed that all eight tested lncRNAs were indeed associated with ribosomes, with seven out of eight having more RNAs associated with ribosomes than with the unbound fraction (Figure 3a and b), suggesting that they are under active translation.

To experimentally validate the expression of these MS‐identified microproteins, two of the nine microproteins (referred to as NONHSAT115127 and NONHSAT092794; 115127 and 092794 for short) that presented in both cells and EVs were chosen for further study. BLAST‐based sequence comparison showed that two homologous sequences (> 94% identity) of 115127 lncRNA encoded microprotein were found in primates (Figure S5), raising the possibility that the 115127 lncRNA encodes an uncharacterized small protein. 092794 lncRNA was derived from the pseudogene of 60S ribosomal protein L21.

To investigate whether the smORFs of these two microproteins are translationally active, we generated a series of constructs. For 115127, a GFPmut—in which the start codon ATGGTG is mutated to ATTGTT—was fused to the C‐terminal of the 5′ untranslated (UTR)‐smORF embedded in the full‐length 115127 transcripts (Figure 3c). Due to the low transfection efficiency of Lipofectamine 3000 Transfection Reagent for glioma cancer cells, the transfection experiments were carried out in 293T cells. The results demonstrated substantial expression of the 115127‐GFP fusion protein in 115127 5′ UTR‐smORF‐GFPmut‐transfected 293T cells (Figure 3d). Similar results were found for 092794‐microprotein (Figure S6). Western blotting analysis using anti‐GFP antibodies also confirmed that the 115127‐GFP fusion proteins exhibited the predicted relative molecular masses in 115127 5′ UTR‐smORF‐GFPmut‐transfected cells (Figure 3e and Figure S7). The MS analysis of 115127‐GFP fusion protein immunoprecipitated from 115127 smORF‐GFP‐transfected cells by anti‐GFP antibody showed 100% sequence coverage (Figure S8). 80.0% of sequence coverage was found for 092794‐microprotein.

To further validate whether microproteins can be actively packaged into EVs, we collected EVs from cells transfected with 115127 5′ UTR‐smORF‐FLAG plasmids. A smORF‐FLAG construct was generated because the GFP tag is larger than the microproteins, which may alter a specific phenotype of the protein (Storz et al., 2014). In the smORF‐FLAG construct, the 3×FLAG tag (22 amino acids) is fused to the C terminus of the 5′ UTR‐smORF in the full‐length lncRNA transcripts (Figure 3f). Substantial expression of the 115127‐FLAG fusion proteins was observed by immunofluorescence staining and Western blotting of 115127 5′ UTR‐smORF‐FLAG‐transfected cells (Figure 3g and h). Similar results were found when using the 092794‐FLAG fusion protein (Figure S6F). Western blot analysis using anti‐FLAG antibodies further confirmed that the 115127‐microprotein‐FLAG fusion proteins had predicted molecular mass in EVs from 115127 smORF‐FLAG‐transfected cells (Figure 3i), signifying that microprotein can be sorted into the EVs.

Finally, we investigated whether these new proteins were potentially functional and in what biological processes they may be involved. 115127‐ microprotein was selected as the lncRNA‐encoded microprotein for further study. Proteins that might interact with 115127‐microprotein were co‐immunoprecipitated (co‐IP) from 115127 smORF‐GFP‐transfected cells by anti‐GFP antibodies and underwent SDS‐PAGE and MS (CoIP‐MS) analysis. SDS‐PAGE analysis revealed that a different profile of protein bands was observed in the co‐IP fractions derived from 115127 smORF‐GFP‐transfected cells compared to GFP‐transfected cells (Figure 3j). MS analysis also showed that multiple proteins, such as PRDX1, PRDX2, PRDX4, MAPK1 and XPO1, were found to be significantly upregulated (Fold change ≧4.0, *P* < 0.05) in the co‐IP fraction derived from the 115127 smORF‐GFP‐transfected cells compared to the GFP‐transfected cells (Figure S9 and Table S3). Interestingly, bioinformatic analysis further demonstrated that these proteins can form a protein‐protein interaction network (Figure 3k), which could be associated with the process of redox stress based on Gene Ontology (GO) analysis. Previous studies have shown that PRDXs are oxidoreductases, capable of scavenging peroxides with reducing equivalents from the thioredoxin‐thioredoxin reductase system. This indicates that PRDXs not only function as a defence against redox stress but also directly support the activation of several protein tyrosine phosphatases, suppressing kinase‐mediated signalling (Firczuk et al., 2014). As such, our results suggest that the function of 115127‐microprotein may be involved in the process of redox stress. However, further studies are needed to fully understand function of the 115127‐microprotein.

### 2.3 Identification of microproteins in plasma EVs from healthy donors

**FIGURE 4:**
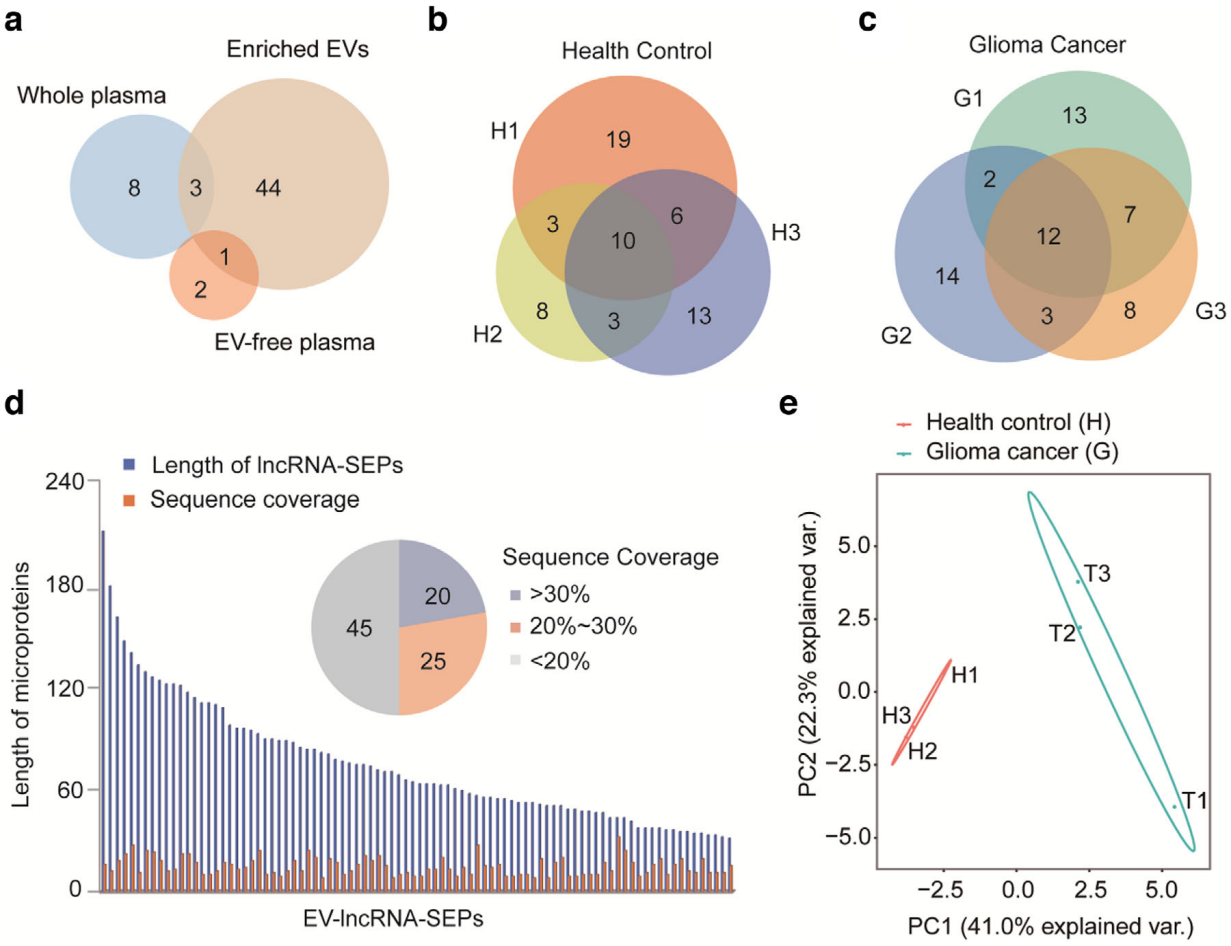
Identification of microproteins from plasma EVs. (a) Microproteins identified in enriched EVs, EV‐free plasma and whole plasma from healthy donors. (b) and (c) Reproducibility of the identification of microproteins from three replicates of circulating EVs from healthy donors and glioma cancer patients, respectively. (d) Length distribution and sequence coverage of microproteins that presented in EVs from either the healthy donors or glioma patients. The values in the sequence coverage pie chart refer to the number of individual microproteins identified. (e) Principal component analysis (PCA) demonstrated clear clustering of the plasma samples from healthy donors and those from glioma cancer patients

Given that microproteins are abundant in EVs, we speculated that microproteins could be detected in circulating EVs derived from human blood. To test this assertion, EVs derived from the plasma of three healthy donors were purified and characterized (Figure S10) and the contained microproteins were subjected to shotgun MS analysis. Whole plasma also underwent microprotein analysis after organic solvent‐based precipitation, and EV‐free plasma—depleted of EV and high abundant proteins by 30KD MWCO—was also subjected to microprotein analysis. Using the stringent criteria for the confident identification of microproteins mentioned above, 56 unique peptides corresponding to 48 microproteins were identified from human plasma EVs, while only three and 11 microproteins were detected in the EV‐free plasma and the whole plasma, respectively (Figure 4a and Table S4). Some degree of reproducible identification can be inferred, since 22 of the microproteins were detected in at least two healthy donors (Figure 4b). These results suggest that the circulating microproteins are primarily associated with plasma EVs, a property that might be attributed to a protective or enrichment effect of the EVs.

### 2.4 Identification of microproteins in plasma EVs from glioma patients

Plasma EVs from cancer patients often carry molecular signatures comprising effectors of different stages of tumour progression. Such signatures can provide valuable biomarkers with potential diagnostic or prognostic value (Barile & Vassalli, 2017). To determine whether EV microproteins could be used as a potential biomarker for cancer detection, we explored the expression profile of plasma EV microproteins of glioma cancer patients and compared them to healthy donors. As done with the healthy control, the plasma from three glioma cancer patients was collected for EV extraction followed by shotgun MS analysis. MS results showed that 73 unique peptides corresponding to 64 microproteins were identified in plasma EVs of glioma patients, with 25 common to at least two glioma patients (Figure 4c and Table S5). Length distribution analysis of the EV microproteins from all donors found that most had a length around 100 aa (Figure 4d). Moreover, ∼32.1% of these microproteins have sequence coverage above 30% (30.2%–95.0%), suggesting high confidence in microprotein identification. We further performed microprotein validation using synthetic peptides and comparing their MS^2^ spectra with that of the tryptic peptides in the selected microproteins. Our results demonstrated that 12 out of 13 selected peptides corresponding to 12 microproteins strongly matched synthetic peptides (Figures S11 and S22).

Most strikingly, the expression profile of EV microproteins of glioma patients and health donors were diverged. Principal component analysis (PCA) distinguished glioma cancer patients from healthy donors (Figure 4e). Both principal component 1 (PC1) and principal component 2 (PC2) separated the plasma samples of healthy donors from that of the glioma cancer patients. Specifically, compared to healthy subjects, 17 microproteins s were missing and 19 new microproteins were detected in cancer patients (Table 1).

**TABLE 1:** List of microproteins specifically identified in EVs from glioma cancer patients

ID^a^	CHR^b^	Start site	End site	Length	Sequence
NONHSAT108542	chr6 (+)	29693230	29697128	35	MCTHDFITGHWMLIFIRSAGAQDTTLLPTGRNPLH
NONHSAT040552	chr14 (−)	106775156	106775618	37	TTSVKGRFTISRDDSKSITYLQMNSLRAEDTAVYYCA
NONHSAT051106	chr15 (−)	101040452	101069470	37	GCHPVASPHVTDCPQSEILTRNFGGWVSEGISGLTQR
NONHSAT071811	chr2 (+)	75711197	75714060	41	MGMWRTFVSSSGIVNAPITALSKQAAGLYQSAGCGWGQIRE
NONHSAT072293	chr2 (−)	89170774	89171212	43	KLENPPKLLIYAASSLPSGVPSRFSGSRSGTHFTHSHHQEPAT
NONHSAT072318	chr2 (+)	89929700	89930202	54	TISSSLAGYQWKPGQALRLLIHGASTRTTNVPAWWSGSGFGENFSLIISRLEHE
NONHSAT083836	chr22 (+)	22915634	22915927	62	KENGTLVTKGIETTTPSTQSNNNYAASSYLSLTPEQWKSHRSYSCQVTHKESTMEKTMAHAE
NONHSAT028900	chr12 (−)	57633328	57634498	63	GEGKPRRGGGAGWEWAYDPCPKPGQEPGRRGPRRRALCINRWRGRLCLTRSLTAQLPAPLLSG
NONHSAT032697	chr13 (+)	28582866	28583699	71	QGFLLRLCHDVGKREVVLTQGTEGVLAVAGGAGAAAHLRVKTTLERPSSLKFDIRIYIREFPTEAICSAGR
NONHSAT083819	chr22 (+)	22762293	22762516	73	YELTQPPAVSVSPGQTARISCSGDVLRDNYADWYPQKPGQAPVLVIYKDGERPSGIPERFSGSTSGNTTALTI
NONHSAT072624	chr2 (+)	97355058	97355335	87	MTQPPSSLSASVGDSVTITCRASQSFTNQLAWYQQKPGKAPKLLIYRVSSLQTGVPSLFSGSESGTDFTLTISSLQPDDVATYYCQQ
NONHSAT040529	chr14 (−)	106586375	106586826	88	GLVQPGGSLRLSCAASGFTFSSSWMHWVCQAPEKGLEWVADIKCDGSEKYYVDSVKGRLTISRDNAKNSLYLQVNSLRAEDMTVYYCV
NONHSAT030405	chr12 (−)	104030743	104032248	89	MKGVGAGDKMAKMKMGATGPHLQLQLKMSTDVVSVTCAGGRGRGTRWAGRVLLGTVSSPPAFTSWPCPGQPQLPWLCAPSPHPPETAWA
NONHSAT141981	chr16 (+)	31962087	31974361	95	EVQLVESGGGLVQPGGSLRLSCAASGFTFSNRSTHWVRQAPGKGLEWVGHSVSKKKKKKKKKKKKGRFTISRDDSKNTLYLQMNSLKTEDTAVYY
NONHSAT072603	chr2 (+)	97059944	97060389	107	MRIPAQLLAFLLLCLPGKEGEHWEMTQPPSSLSASVGDRVTVSCQASQSIYNYLNWYQQKPGKAPKFLTYRASSLQRGMPSQFSGSGYGRDFTLTVSSLQPEDFATY
NONHSAT040524	chr14 (−)	107034726	107034966	110	ISCKGSGYSFSTYWVGWVRQIPGKGLEWMAIIYPSDSDTRYSPSFQGQVTISADKSISTAYLQWSSLKASDTAMYYCARHGYLSSGKGYFDYWGQGTQVTVSSGSASAPT
NONHSAT142116	chr16 (−)	33938336	33938799	113	AEFSLLLFLKVSSVRCSWWSLPEALVQPGGSLRLSCAASGFTCSNAWMSWVRQAPGKGLEWVGRIKSKANGGTTDYAAPVKGRFTISRVDSKNTLYLQMNSLKTEDTAVYYCT
NONHSAT040427	chr14 (−)	105864215	106268900	125	VQLLESGGGLVQPGGSLRLSCAASGFTFSSYAMSWVRQAPGKGLEWVSAISGSGGGTYYADSVKGRFTISRDNSKNTLYLQMNSLRAEDTAVYYCAKDRAPYSSSFDYWGQGTLVTVSSRSASAP
NONHSAT011749	chr10 (−)	22435424	22437929	210	GVRWIQQNKHHKKIICERRRRCCENLVPETVSEQQKNRRKPKEHDPDGDGGGARGQHCHLPPTPGLKSERRLGGHKCRLLLSFAEAEKSPSGARRASRGKRAVTIPTGRPQRSLSADKAASKGAPRLPSHHVPFGRRKRKRPLPVRQPDNSGGQAAPRQHLCFPRAGKRSLRIPQKPEADTTETRGRSSRSDVYSGRERGGHLRLGEAAG

^a^ NONCODE transcript ID.

^b^ Chromosome (Strand).

## 3 DISCUSSION

Advancements in technology over the past few years have led to the discovery of numerous biologically relevant microproteins from a variety of species, and we expect even more such peptides to be discovered. However, the mechanisms and modes of action that underlie their roles are yet to be fully characterized. In the current study, we discovered and demonstrated for the first time the presence of abundant microproteins in cell culture‐derived and circulating EVs. Considering significant divergent roles of microproteins in many fundamental biological processes, where some also show important relationships with pathogenesis, the EV‐mediated transfer of microproteins might represent a previously unknown mechanism of cell‐to‐cell communication.

Our improved discovery rate of microproteins can be partially credited to our newly constructed in‐house microprotein reference database, which completely collected the putative smORFs from the NONCODE database by combining the six‐frame translation mode and ORFfinder. Although the expanded microprotein reference database may result in an elevated threshold to control false discovery rate (FDR) (Jeong et al., 2012), it enabled us to data‐mine substantially more microproteins from cell lines and tissues once stringent identification criteria were employed. Moreover, the experimental evidence acquired from translation, mass spectrometry, antibodies and bioinformatics further validated the existence of these microproteins.

Our results demonstrated that 84 and 29 microproteins were identified from glioma cells and their EVs, respectively, with nine microproteins identified in both cells and EVs. This suggests that the release of specific microproteins in or with EVs may be actively regulated. However, the localization of the identified microproteins inside or on the surface of EVs has not been defined in current study, which could be determined by a combination of proteinases (e.g., proteinase K (PK) or trypsin/Lys‐C) treatment and LC‐MS/MS analysis. Moreover, it is noteworthy that in cancer EV contents are thought to regulate diverse pivotal processes and functions of tumour cells such as growth, proliferation, survival, migration, neo‐angiogenesis, immunomodulatory functions and anti‐cancer drug resistance (Ma et al., 2017). Therefore, the tumour‐derived EV microproteins might be of potential to be used to modulate the function of recipient cells, and thus, reveal the pathophysiological status of tumour cells.

In human blood plasma, 48, 11 and 3 microproteins were identified from the purified EVs, whole plasma and EV‐free plasma, respectively. These results suggest that there is an enrichment of circulating microproteins in EVs. However, it is worth noting that fewer microproteins were detected in whole plasma could partially due to high overall protein levels, which could mask the microprotein detection to a certain extent. Meanwhile, some of the identified microproteins might not be EV‐associated but rather copurified contaminants. Nonetheless, these results indicate that cell‐to‐cell communication by microproteins could occur at a distance by the trafficking of EVs through systemic circulation. In fact, considering the low abundance of microproteins in cells, EVs would be a good choice for the transportation and/or exchange of microproteins between distant cells. Accumulating evidence has suggested that EVs can be a reservoir of novel biomarkers for cancer (Ding et al., 2020; Lane et al., 2019). Furthermore, EVs could protect microproteins from proteolytic enzymes, thereby keeping their integrity and functionality intact.

Most importantly, our preliminary data showed that the circulating microproteins enriched in plasma EVs are able to distinguish patients with glioma cancer from healthy donors, suggesting that circulating microproteins in EVs might have potential diagnostic application in identifying patients with glioma. However, a larger cohort of patients with glioma cancer and healthy controls are still needed to further validate the potential of the circulating microproteins identified in EVs as new biomarkers for glioma diagnosis. Nonetheless, this finding could provide new opportunities to find new biomarkers for glioma diagnosis and prognosis. Glioma is the most common malignant primary brain tumour in the central nervous system (CNS), and the lack of reliable non‐invasive diagnostic and prognostic methods is one of the main reasons for its high mortality (Goodenberger & Jenkins, 2012). Moreover, there are three distinct advantages in using EV microproteins as biomarkers for glioma diagnosis or prognosis: (1) microproteins in EVs can readily cross the blood–brain barrier and enter into circulation along with EVs (Saint‐Pol & Gosselet, 2020), (2) microproteins would be more functional and integral compared to that travel outside EVs, as EV microproteins are protected from the ubiquitous presence of proteolytic enzymes (Cheng et al., 2014), and (3) the expression of many microproteins is tissue specific (unpublished data), which might reflect the ongoing physiological and pathological state of an individual. Therefore, it would be of great benefit to further prove the feasibility of using EV microproteins as biomarkers for the diagnosis or prognosis of glioma and potentially other cancers.

Last but not least, with regard to the characteristics of microproteins, 53.6% of identified microproteins had an unknown start codon (Non‐AUG) and less than 15% initiated with AUG. Similar results were found from circulating microproteins identified from either healthy donors or patients with glioma cancers. This could partially explain why previous studies have missed the identification of these new microproteins, as Non‐AUG initiation make the novel microproteins less probable and thereby excluded from most MS‐based reference databases. Although it was long thought that eukaryotic translation generally initiates at an AUG start codon, thousands of previously unannotated initiation events have now been identified in mouse embryonic stem cells, ∼60% of which initiate at a non‐AUG start codon (Andreev et al., 2017; Ingolia et al., 2011). These non‐AUG initiation events are not simply errors but are instead used to generate or regulate proteins with key cellular functions (Kearse & Wilusz, 2017). The mis‐regulation of non‐AUG initiation events has been found in multiple human diseases, including cancers and neurodegeneration and the modulation of non‐AUG usage may represent a novel therapeutic strategy (Brar & Weissman, 2015; Chu et al., 2016). As such, microproteins identified in current study may impose certain impacts on cellular states, and the function of these microproteins could be further uncovered in conjunction with improvements of effective genome‐engineering strategies.

Overall, our results suggest that microproteins might participate in intercellular information exchange based on the EV‐mediated release, transport or uptake. Further elucidating the function of microproteins in normal cells and their deregulation in cancer cells might provide crucial information to understand cancer biology.

## 4 MATERIALS AND METHODS

### 4.1 Cell culture

Glioma cancer cells and HEK‐293T cells were cultured in Dulbecco's modified Eagle's medium (DMEM) (Gibco, CA, USA) supplemented with 10% fetal bovine serum (FBS) and antibiotics (Gibco, CA, USA). EVs in the FBS were depleted by filtration through a 0.22‐µm filter and ultracentrifugation at 110,000 *g* for 16 h.

### 4.2 Plasma collection

The plasma samples for healthy donors used in this study were provided by Medical College, Henan University, Kaifeng, Henan Province, China. Written informed consent was obtained from participants. This study was approved by the Ethical Committee of Medical Science and Research, Medical College, Henan University (HUSOM‐2018‐388). The plasma samples of glioma patients used in this study were provided by Capital Medical University Sanbo Brain Hospital. The individual patient data has been attached in the Table S6. The research protocol was approved and supervised by Capital Medical University Sanbo Brain Hospital. All procedures performed in studies involving human participants were in accordance with the ethical standards of the institutional and with the 1964 Helsinki declaration and its later amendments or comparable ethical standards. Whole blood was collected into ethylenediaminetetraacetic acid (EDTA)‐anticoagulated tubes and separated by centrifugation into plasma and cellular fractions. The plasma samples were stored at ‐80°C.

### 4.3 Extracellular vesicles isolation

The isolation and characterization of EVs from cell culture or plasma were performed with adherence to MISEV2018 guidelines (Théry et al., 2018). The relevant data of our experiments are accessible in EV‐TRACK knowledgebase (Van Deun et al., 2017) with identifier EV210109 and the last name of the first author (Cai).

Briefly, to isolate cell culture‐derived EVs, cells were cultured in EVs‐depleted DMEM medium for 48 h. The medium was collected and immediately underwent differential centrifugation at 500×*g* for 5 min, 2000×*g* for 20 min at 4°C to remove cells and debris. The medium was then ultracentrifuged at 100,000×*g* for 70 min at 4°C using a Beckman ultracentrifuge with Type 45Ti rotor. The supernatant was discarded, and the pellets were resuspended in 70 ml PBS followed by re‐centrifugation. The final pellet was suspended in PBS and stored at ‐80˚C for further analysis.

For plasma samples, EVs were isolated using asymmetrical flow field‐flow fractionation (AF4) technology with an Eclipse 3 system (Wyatt Technology Europe GmbH, Dernbach, Germany) as previously described (Wu et al., 2020). The system contains a HPLC isocratic pump (Agilent Technologies, Palo Alto, CA, USA), a manual injection valve and 500 µl stainless steel sample loop, a UV detector at a wavelength of 280 nm (Agilent Technologies, Palo Alto, California) and a MALS detector with 18 scattering angles from 14.4° to 163.3° (DAWN HELEOS II, Wyatt Technology) at 658 nm. The separation channel was a trapezoidal shaped Mylar (Polyethylene‐terephthalate) spacer with a thickness of 350 µm above a regenerated cellulose membrane (10 kDa, R7PA81151, SUPERON). The tip‐to‐tip length of the channel was 152 mm and the initial triangle channel breadth of 21.5 mm was decreased to a final breadth of 3 mm. PBS buffer (pH 7.4) was filtered with a 0.22 µm filter membrane and used as mobile phase for all separation. All data acquisition and processing were performed using Astra software (Version 5.3.4.20, Wyatt Technology). All AF4 experiments were performed at room temperature. The total flow rate was maintained at 2.7 ml/min for 9 min for sample injection and simultaneous focusing/relaxation. Thereafter, the two valves were rotated so that all flows entered the channel inlet for separation at flow rate conditions (outflow rate = 4 ml/min and crossflow rate = 3 ml/min). Exosome fractions were collected during AF4 separation for proteomic analysis.

### 4.4 Isolation of microproteins from EV‐free plasma and whole plasma

The isolation of microproteins from EV‐free plasma was achieved by ultrafiltration (UF) using a 100‐kDa molecular weight cut‐off (MWCO) Amicon Ultra‐15 centrifugal filter unit (Millipore). Briefly, 100 µl of plasma was diluted with 900 µl of 2% acetonitrile (ACN) and 0.1% formic acid (FA) in water. After centrifugation at 20,000 *g* for 20 min at 4℃ in a bench‐top centrifuge, the supernatant was filtered through a 30 kD MWCO filter (Millipore) and the flow through was collected and evaporated until dry by vacuum centrifugation at 4℃. The pellet was then dissolved in 8 M urea/ 100 mM NH_4_HCO_3_ and stored at ‐80°C.

For isolation of microproteins from whole plasma, the low molecular weight fraction of plasma was collected using a sequential precipitation and de‐lipidation (1) method developed for the efficient enrichment of the low molecular weight proteins/peptides from plasma based on methyl‐tert‐butyl ether/methanol/water systems (Li et al., 2020). Briefly, a single‐phase solvent system MTBE/methanol/water (5:3:1, v/v) and a two‐phase solvent system MTBE/methanol/water (5:1:1, v/v) were applied for sequential precipitation and de‐lipidation of plasma. A total of 50 µl of deionized water and 250 µl of MTBE were added to 50 µl of plasma in a tube and vortexed. Then, 150 µl of methanol was added to the MTBE/methanol/water ratio of 5:3:1 (v/v) and incubated for 30 min at 4°C for precipitation. After centrifugation at 21,000 *g* for 30 min at 4°C, the supernatant was carefully collected into a new tube and 500 µl of MTBE and 100 µl of deionized water were added for a new MTBE/methanol/water ratio of 5:1:1 (v/v). After intensive mixing, the sample solution formed two phases. The upper phase contained lipids and was discarded after centrifugation at 1000 *g* for 10 min at 4°C. The lower phase contained LMWPs and was collected and lyophilized under vacuum for LC‐MS/MS analysis.

### 4.5 Sample preparation for transmission electron microscopy

EVs were separated by ultracentrifugation and the pellet resuspended in PBS. One drop of the resuspended EVs was put onto a copper mesh for 1 min. The fluid was then absorbed from the edges of the copper mesh with filter paper. The copper mesh was stained with 1% uranyl acetate in ddH_2_O for 1 min. The sample was then dried for 5 min under incandescent light.

### 4.6 Nanoparticle tracking analysis (NTA)

The EVs isolated from glioma cells and plasma were analysed by NTA to determine vesicle concentration and size distribution using Nanosight NS300 (Malvern, UK) equipped with blue laser (488 nm) and sCMOS camera. The samples were diluted in filtered (0.1 µm) PBS to obtain the optimal detection concentration of 10^6^–10^9^ particles/ml, and three 60 s videos were recorded using camera level 10. The data were analysed using NTA software 3.4 Build 3.4.003 with the detection threshold 4.

### 4.7 Putative microprotein database

To construct a comprehensive microprotein database covering all putative small ORFs from lncRNA transcripts for MS–based identification of microproteins, lncRNA transcripts were downloaded from the NONCODE database, which possesses the most complete collection and annotation of non‐coding RNA. All transcripts were scanned by ORFfinder and six‐frame translation mode to obtain smORFs. ORFfinder scanned smORFs containing Kozak sequences and AUG initiation codons. In six‐frame translation mode, lncRNAs were read in six possible reading frames, three in the forward direction and three in the reverse direction. Each frame resulted in different smORFS containing a start and stop codon that can translate into possible proteins. The combination of six‐frame translation mode and ORFfinder thus allowed our microproteins database to completely cover all putative smORFs from lncRNA transcripts.

### 4.8 In solution digestion and desalting

The cell lysates or EV samples were precipitated with acetone and resolved in 8 M urea with 50 mM Tris‐HCl (pH = 8.0). The samples were reduced by 10 mM dithiothreitol, alkylated by 10 mM iodoacetamide (45 min at RT in the dark), and digested with sequencing‐grade modified trypsin (Promega) at a substrate ratio of 1:50 (w/w) overnight at 37 °C. Then, the peptide sample was desalted with a C18 Oasis HLB cartridge (Waters, USA) and dried in a centrifugal vacuum concentrator (Labconco, MI, USA).

### 4.9 Fractionation of peptides with high pH reverse‐phase HPLC

The concentrated samples were dissolved in high pH fraction buffer A (98% H2O/2% ACN, pH 10, pH adjusted with ammonium hydroxide) and loaded onto an XBridge C18 basic reversed‐phase LC column (150 × 2.1 mm, 3.5 µm particles) (Waters). Peptides were separated in a Rigol L‐3000 LC system (Beijing, China) with a binary buffer system of buffer A and buffer B (98% ACN/2% H2O, pH 10). The gradient of buffer B was set as follows: 5–8% for 5 min, 8–18% for 35 min, 18–32% for 22 min, 32–95% for 2 min, and 95% for 4 min. Eluents were collected every 90 s, and a total of 42 fractions were obtained. These fractions were combined into 10 fractions by merging fractions 1, 11 and 21; fractions 2, 12 and 22; and so on. Then, all the combined fractions were dried in a Benchtop Centrifugal Vacuum Concentrator and stored at ‐20°C for further MS analysis.

### 4.10 LC‐MS/MS analysis

All mass spectrometry data were collected on a Q‐Exactive mass spectrometer (Thermo Fisher Scientific) coupled with an Easy‐nLC system (Thermo Fisher Scientific). The digested peptides were loaded onto a 100 µm id × 2 cm fused silica trap column packed in‐house with reversed phase silica (Reprosil‐Pur C18 AQ, 5 µm, Dr. Maisch GmbH) and then separated on an a 75 µm id × 20 cm C18 column packed with reversed phase silica (Reprosil‐Pur C18 AQ, 3 µm, Dr. Maisch GmbH). The peptides bound on the column were eluted with a 78 min linear gradient. The solvent A and B consisted of 0.1% FA in water and ACN solution, respectively. The segmented gradient was 5–8% B, 8 min; 8–22% B, 50 min; 22–32% B, 12 min; 32–95% B, 1 min; 95% B, 7 min at a flow rate of 300 nl/min.

The separated peptide ions eluted from the analytic column entered the mass spectrometer at an electrospray voltage of 2.1 kV. All MS/MS spectra were acquired in a data‐dependent mode for fragmentation of the 10 most abundant peaks from the full MS scan with 30% normalized collision energy. The dynamic exclusion duration was set at 20 s and the isolation mass width was 2.5 Da. MS spectra were acquired with a mass range of 400–1800 m/z and a resolution of 70,000 at m/z 200. MS/MS resolution was acquired at a resolution of 17,500.

### 4.11 Identification of microproteins

The LC‐MS/MS data were analysed using Proteome Discoverer (Version 1.4, Thermo Fisher Scientific) against the Uniprot KB human database (released on September, 2018), into which 175 commonly observed contaminants and all the reverse sequences were added, as well as by the in‐house putative microprotein database. Search parameters were as follows: trypsin was selected as the enzyme and two missed cleavages were allowed for searching; precursor ions mass tolerance of 10 ppm (monoisotopic mass), fragment ions mass tolerance of 0.02 Da (monoisotopic mass); the cysteine carbamidomethylation was specified as fixed modification; the methionine oxidation was chosen as variable modification. The search results were passed through additional filters before the data was exported. For both annotated protein and microprotein identification, the filters were set as follows: FDR < 1% and significance threshold *P* < 0.05.

To guarantee high confidence identification of microproteins, identified peptides were manually checked via sequence and spectrum and a series of strict criteria were set by following Slavoff, S.A. et al. (Slavoff et al., 2013) and He, C. et al. (He et al., 2018) with slight modifications: (1) Peptide length cannot be less than 6 aa, (2) Spectra must have more than four continuous b‐ or y‐ ions and few impure peaks with high intensity, (3) Spectra must have more than 40% b‐ and y‐ ions coverage, and (4) Peptide with a sequence identical to annotated proteins in UNIPROT database must be excluded. The peptides that passed the filtering conditions were considered as novel peptides and their corresponding microproteins were confirmed as candidates for novel microproteins. The raw data and search result files are available in ProteomeXchange with identifier PXD019486 (Username: reviewer77742@ebi.ac.uk; Password: 4BqwhtiT).

### 4.12 Analysis of synthetic standard peptides for the validation of identified microproteins

To further validate the identification of novel microproteins, 14 standard peptides from 14 microproteins were synthesized by GL Biochem (Shanghai, China) and analysed on the same LC‐MS/MS instrument as above.

### 4.13 Quantitative reverse transcription PCR (qRT‐PCR)

Total RNA was converted into cDNA by reverse transcription using a SuperScript III reverse transcriptase kit (Thermo Fisher Scientific). Gene expression was quantified using TransStart Top Green qPCR SuperMix (TransGen Biotech). The PCR reactions were conducted in triplicates using the Rotor‐Gene Q real time PCR system (Qiagen). Sequences for the gene‐specific primers are listed in Table S7. β‐actin was used as an internal control.

### 4.14 Polysome fractionation and lncRNA quantification

Glioma cells were treated with 0.1 mg/ml cycloheximide (Sigma‐Aldrich) for 10 min at 37 °C, disaggregated with trypsin‐EDTA (0.25%) for 1 min and washed twice with ice cold PBS containing 0.1 mg/ml cycloheximide. Polysome lysis buffer composed of 20 mM Tris HCl (pH 7.4), 3 mM MgCl_2_, 100 mM KCl, 0.1 mg/ml cycloheximide, 0.5% Triton X‐100, 50 U/µl RNasin was used to resuspend cells, followed by 10 min incubation on ice and 3 min centrifugation at 16000×*g* at 4 °C. Sucrose gradients were prepared with BioComp model 108 Gradient Master using 10% and 50% sucrose solutions (sucrose diluted in polysomal buffer containing 20 mM Tris HCl (pH 7.4), 3 mM MgCl_2_ and 100 mM KCl, 0.1 mg/ml cycloheximide and 50 U/µl RNasin). Supernatants were loaded onto 10‐ml continuous 10–50% sucrose gradients and centrifuged at 220000×*g* (SW41 rotor, Beckman) for 150 min at 4 °C. Sucrose gradient fractions were separated using Econo Gredient Pump (Bio‐RAD), and the absorbance was monitored at 254 nm to record the polysome profile. Total RNA was extracted from each fraction by TRIZOL and subjected to Realtime RT‐PCR analysis.

### 4.15 Plasmid constructs

To generate eGFP fusion protein constructs with the microprotein 5′untranslated (UTR)‐smORF C terminus (5′UTR‐smORF‐GFP), the microprotein 5′UTR‐smORF sequences containing the endogenous 5′UTR were amplified using RT‐PCR and cloned into a pEGFP‐N1 vector in which the GFP start codon (ATGGTG) was mutated to ATTGTT (pGFPmut) (Clontech). To generate FLAG fusion protein constructs with the microprotein 5′UTR‐smORF C terminus (5′UTR‐smORF‐FLAG), the microprotein smORF sequences were amplified with the corresponding endogenous 5′UTR using RT‐PCR and cloned into the pECMV_3×FLAG vector (MiaoLing Plasmid Sharing Platform). The primers used in this study are listed in Table S8.

### 4.16 Immunofluorescence staining

293T cells were transfected with microprotein 5′UTR‐smORF‐GFPmut vectors for 24 h, and GFP fluorescence was directly imaged and recorded. 293T cells transfected with microprotein 5′UTR‐smORF‐FLAG was plated on glass coverslips. These cells were fixed with 4% paraformaldehyde, permeabilized with 0.1% Triton X‐100, incubated with anti‐FLAG antibodies (ABClonal, AE037, 1:250) and subsequently incubated with Goat Anti‐Mouse IgG H&L (Alexa Fluor 555) (Abcam, ab150118, 1:1000) or Cy3‐conjugated secondary IgG antibodies. Cellular nuclei were stained with DAPI (Solarbio, C0065).

### 4.17 Anti‐microprotein antibody preparation

Peptide synthesis and preparation of anti‐ microprotein antibodies was performed by Genescripts (Nanjing). Briefly, KLH‐coupled peptides GGARGPAPAEGPAPC‐Cys (NONHSAT115127) and IHKKGDIVDIKGMDC‐Cys (NONHSAT092794) were synthesized, and polyclonal antibodies against the corresponding peptides were obtained from inoculated rabbits. Antibodies were purified using affinity chromatography on columns containing the corresponding peptides.

### 4.18 Western blotting

Cells were suspended in lysis buffer (25 mM Tris‐HCl (pH7.5), 150 mM NaCl, 1 mM EDTA, 1% Triton X‐10050 mM Tris‐HCl pH 8.0, 1% SDS, 1 mM EDTA, 5 mM DTT, 10 mM PMSF, 1 mM NaF, 1 mM Na3VO4, and protease inhibitor cocktail), and then denatured in boiling water for 5 min. The cellular lysates were centrifuged at 12,000 rpm for 30 min. The protein concentration was determined by BCA assay. For EVs protein characterization, 15 µg of proteins were separated on 4–20% Tris‐Glycine Gel (Novex WedgeWell, Invitrogen) and transferred onto PVDF membranes. Western blotting was conducted with the following primary antibodies at appropriate dilution: CD9 (Abcam, ab92726, 1:2000); CD63 (Abcam, ab134045, 1:1000); CD81 (Abcam, ab109201, 1:1000); ALIX (Abcam, ab186429, 1:1000); Annexin A1 (Abcam, ab214486, 1:2000); TSG101(Abcam, ab125011, 1:1000); HSP90 (Abcam, ab34909, 1:1000); ApoA1 (Abcam, ab52945, 1:20000); ApoB (Abcam, ab20737, 1:1000, cross‐reacts with ApoB48 and B100). For the detection of microprotein‐FLAG fusion proteins, whole cell lysates and tissue lysates were separated using 10% Tricine‐SDS‐PAGE, and then electroblotted onto a polyvinylidene fluoride (PVDF) membrane. Western blotting was performed using anti‐FLAG (1:5000) or anti‐microprotein (1:5000) antibodies. For the detection of microprotein‐GFP fusion proteins and β‐tubulin, whole cell lysates were separated using 12% NuPAGE Bis‐Tris Gel (Invitrogen, NP0346BOX) and 10%–12.5% Tris‐SDS‐PAGE, and western blotting was performed using anti‐GFP (ABclonal tech, AE012,1:5000), and β‐tubulin (Yeasen tech, 30303ES50,1:10000) antibodies.

### 4.19 Co‐immunoprecipitation and mass spectrometry

Whole cell lysates from 293T cells stably expressing NONHSAT115127 smORF‐GFP were prepared using lysis buffer (25 mM Tris‐HCl (pH7.5), 150 mM NaCl, 1 mM EDTA, 1% Triton X‐100, and protease inhibitor cocktail). Co‐IP was conducted using GFP agarose beads (KT health, KTSM1301). The microprotein‐GFP complexes were separated and the gels stained with silver. Three independent experiments were performed. The differential gel bands and their corresponding negative gel bands were excised and underwent in‐gel trypsin digestion. The extracted peptide mixtures were dissolved in a buffer containing 0.1% FA and 2% ACN and analysed using nano‐LC‐MS/MS as described above.

### 4.20 Bioinformatics analysis

Evolutionary conservation of microproteins was analysed by running a sequence similarity search between the amino acid sequences of identified microproteins with that from other species, including Bacteria, *S. cerevisiae*, *C. elegans*, Fruitfly, Zebrafish, Chicken, Mouse, Rat and Gorilla, with the BLAST (Basic Local Alignment Search Tool) program in Uniprot database (https://www.uniprot.org/). The percent identity (% identity) was used to indicate the similarity/identity of the query sequence to database sequences. The % identity of each microprotein sequence to that hits with the highest score from a particular species in the database was retrieved for further analysis.

Volcano plot employed to visualize the expression levels of proteins identified in the co‐IP fraction derived from the NONHSAT115127 5 ’UTR‐smORF‐GFP‐ and GFP‐transfected cells was performed by ggplot2 package (https://ggplot2.tidyverse.org/). The protein‐protein interaction networks were analysed using the STRING v10 system (https://string‐db.org/) and Adobe Illustrator CC based on the proteins that were detected in all three replicates and significantly upregulated (Fold change ≧4.0, *P* < 0.05) in the co‐IP fraction derived from the NONHSAT115127 5 ’UTR‐smORF‐GFP‐transfected cells compared to the GFP‐transfected cells. The Database for Annotation, Visualization and Integrated Discovery (DAVID) was used for GO analysis of the proteins upregulated in the co‐IP fraction derived from the NONHSAT115127 5′UTR‐smORF‐GFP‐transfected cells.

Principal Component Analysis was performed based on the raw area intensity of microprtein over the Total Area Sum Intensity of all microproteins that identified at least twice within each group by ggbiplot, a R package tool for visualizing the results of PCA analysis (http://github.com/vqv/ggbiplot).

## CONFLICT OF INTEREST

The authors declare that they have no competing interests.

## AUTHOR CONTRIBUTIONS

Tanxi Cai, Qing Zhang, Hongwei Zhang and Fuquan Yang designed the research. Tanxi Cai, Qing Zhang, Bowen Wu, Jifeng Wang, Na Li, Tingting Zhang, and Zhen Fan performed the research. Jianjun Luo and Runsheng Chen provided the putative microprotein database and valuable discussion. Weihai Ning and Xiangqian Guo provided plasma samples. Tanxi Cai, Qing Zhang, Bowen Wu, Zhipeng Wang, Xiangqian Guo, Xiang Ding, Zhensheng Xie, Lili Niu and Xiaowei Chen analyzed the data. Tanxi Cai, Qing Zhang, and Fuquan Yang drafted the manuscript with input from the other authors. All authors approved the final submitted version of this paper.

## Supporting information

Supplementary information

Supplementary information

Supplementary information

Supplementary information

Supplementary information

Supplementary information

Supplementary information

Supplementary information

Supplementary information

## Data Availability

All data needed to evaluate the conclusions in the paper are present in the paper and/or the Supplementary Materials. Additional data related to this paper may be requested from the authors.
