# Supplementary material for: LncRNA‐encoded microproteins: A new form of cargo in cell culture‐derived and circulating extracellular vesicles
Source: J Extracell Vesicles. 2021 Jul 12;10(9):e12123. doi: 10.1002/jev2.12123 (PMC8275822; doi:10.1002/jev2.12123)
Supplement: Supplementary file 1 — Supplementary information [file JEV2-10-e12123-s008.pdf]

Supplementary Figures

Figure S1. The representative MS/MS spectra for microproteins identified from glioma cancer cells and EVs.

Figure S2. Validation of the identified microproteins from glioma cells and EVs.

Figure S3. Expression levels of microprotein-encoding lncRNA transcripts in different tissues.

Figure S4. Expression of several microprotein-encoding lncRNAs in glioma cells.

Figure S5. BLAST-based sequence comparison.

Figure S6. Experimental validation for the presence of NONHSAT092794-microprotein in 293T cells.

Figure S7. Full size images of Western blot for detection of microprotein-GFP- or –FLAG-fusion proteins.

Figure S8. The MS/MS spectra of peptides derived from NONHSAT115127-microprotein.

Figure S9. CoIP-MS analysis of proteins potentially interacted with NONHSAT115127-microprotein based on anti-GFP antibodies.

Figure S10. Characterization of plasma EVs isolated by AF4 system.

Figure S11-S22. Validation of MS-identified microproteins in plasma either from healthy donors or glioma cancer patients.

>NONHSAT036368

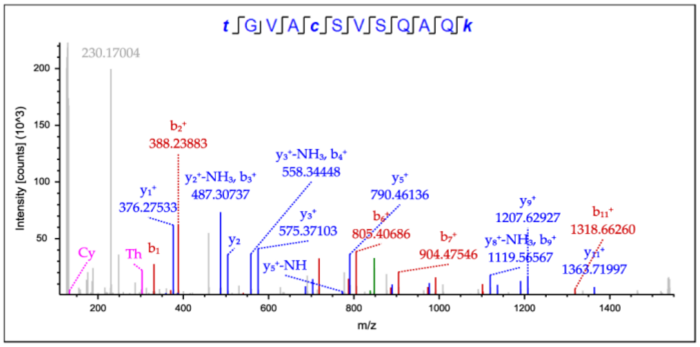

>NONHSAT136922

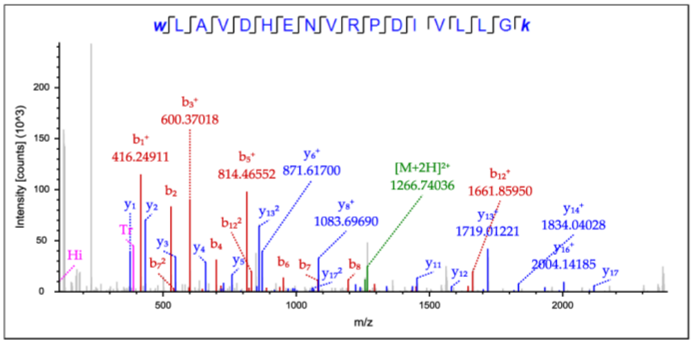

>NONHSAT015982

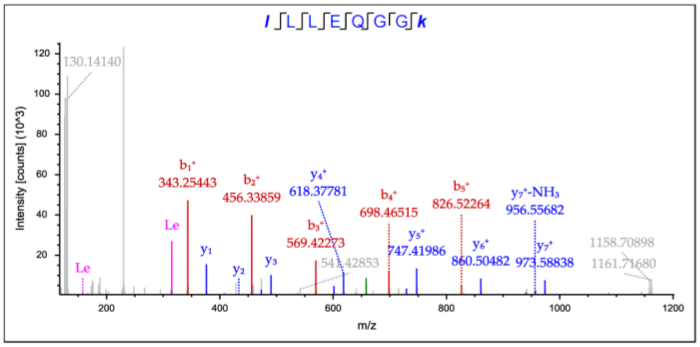

>NONHSAT040410

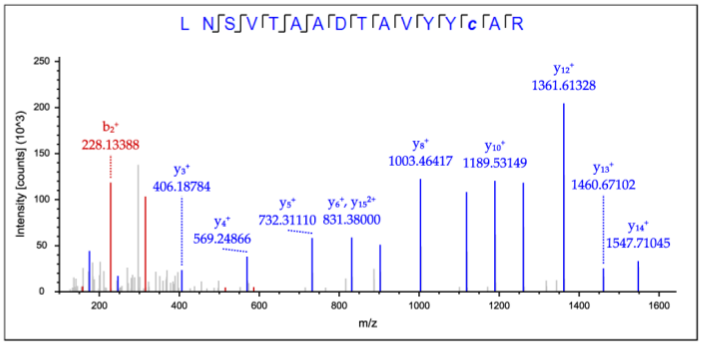

>NONHSAT040410

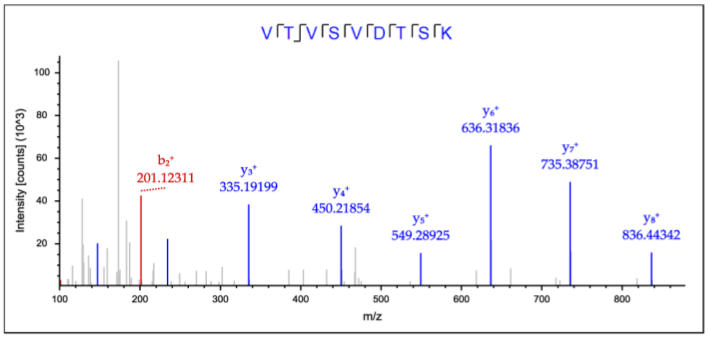

>NONHSAT040428

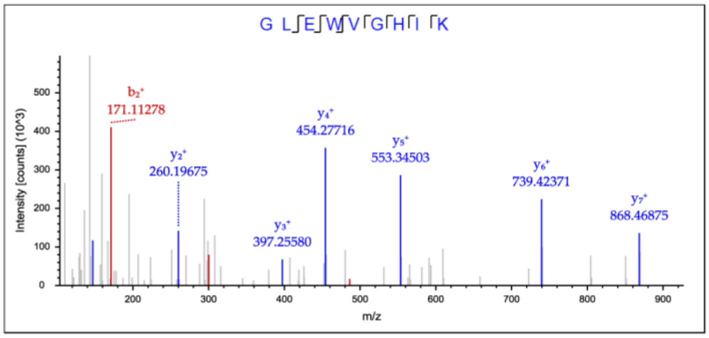

>NONHSAT040428

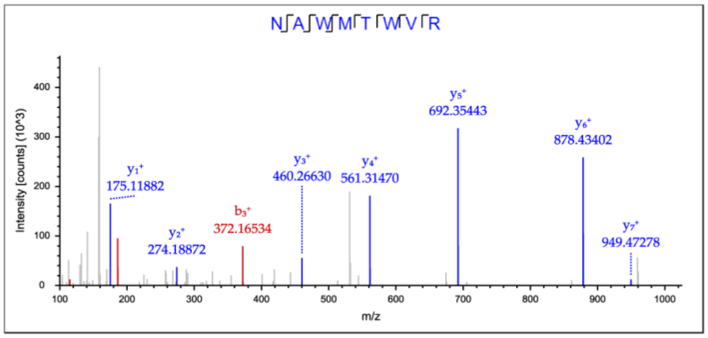

>NONHSAT047368

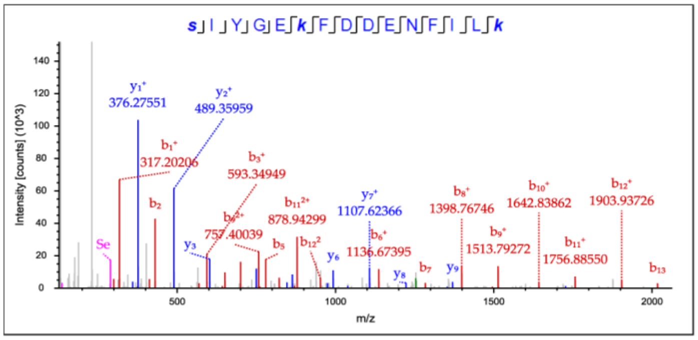

Figure. S1. The representative MS/MS spectra for microproteins identified from glioma cancer cells and EVs.

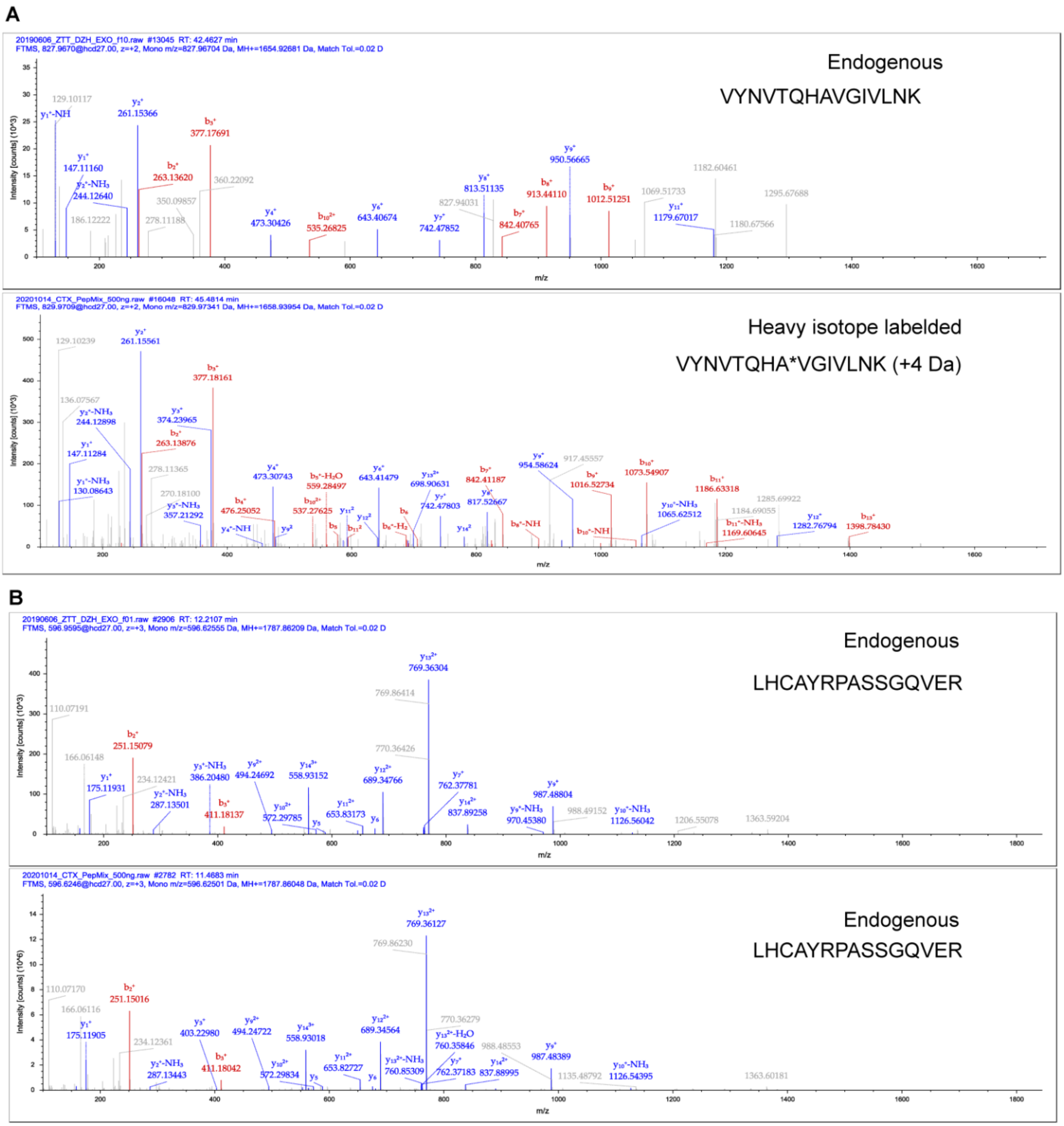

Figure S2. Validation of the identified microproteins from glioma cells and EVs. (A) The interpreted MS/MS spectra of the endogenous peptide VYNVTQHAVGIVLNK and the heavy isotope labeled peptide VYNVTQHA*($^{13}C_3{}^{15}N$)VGIVLNK. (B) The interpreted MS/MS spectra of the endogenous and synthetic peptide of LHCAYRPQSSGQVER.

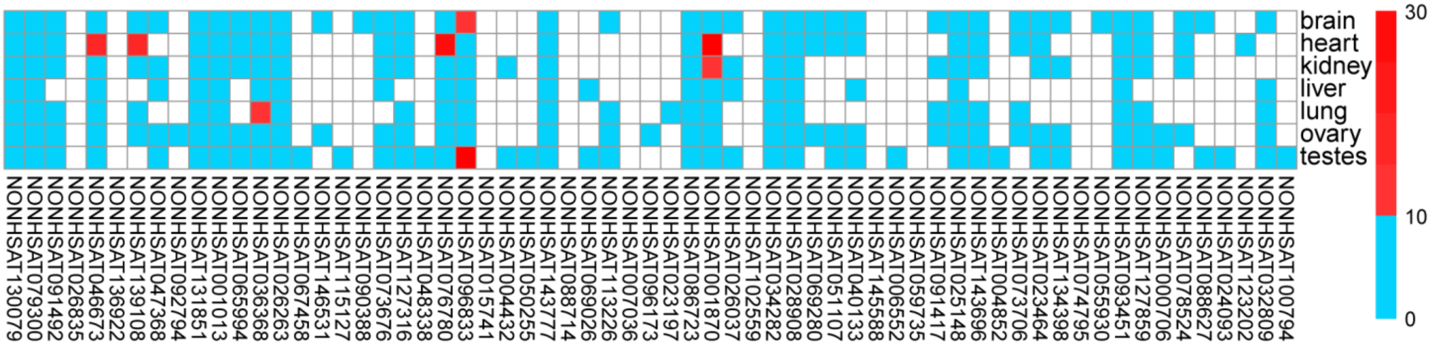

Figure S3. Expression levels of microprotein-encoding lncRNA transcripts in different tissues. Low expression levels of the new microprotein-encoding lncRNA transcripts in different tissues (< 10 RPKM) were primarily observed. The expression levels of microprotein-encoding lncRNA transcripts in different tissues was retrieved from the the NONCODE (http://www.noncode.org/) database.

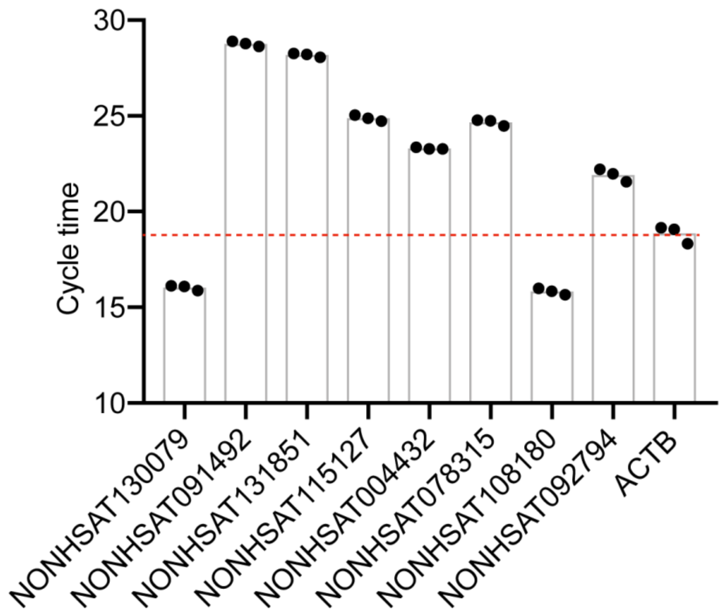

Figure S4. Expression of several microprotein-encoding lncRNAs in glioma cells. Quantitative RT-PCR was employed to evaluate the expression of several microprotein-encoding lncRNAs in glioma cells. The mRNA of actin (ACTB) was used as control.

**Homo sapiens**	**1** GSALILHEGEVTVKEDKINALIKAAGVNVEPFWTGLFAKALANVDMGSLICNG **53**
**Gorilla gorilla gorilla**	**1** CSALILHEGEVTVKEDKINALIKVAGVNVEPFWTGLFAKALANVDMGSLICNG **53**
**Pan troglodytes**	**1** CSALILHEGEVTVKEDKINALIKVAGVNVEPFWTGLFAKALANVDMGSLICNG **53**

**Homo sapiens**	**54** GARGPAPAEGPAPSTNAVPAEEKK------------- **77**
**Gorilla gorilla gorilla**	**54** GARGPAPAEGPAPSINAVPKRESEESKEDMGFGLFD **97**
**Pan troglodytes**	**54** GARGPAPAEGSAPSTNAVPKRESEESKEDMGFVLFD **97**

Figure S5. BLAST-based sequence comparison. Two homologous sequences of NONHSAT115127 encoded microprotein were found in primates but not in any other species.

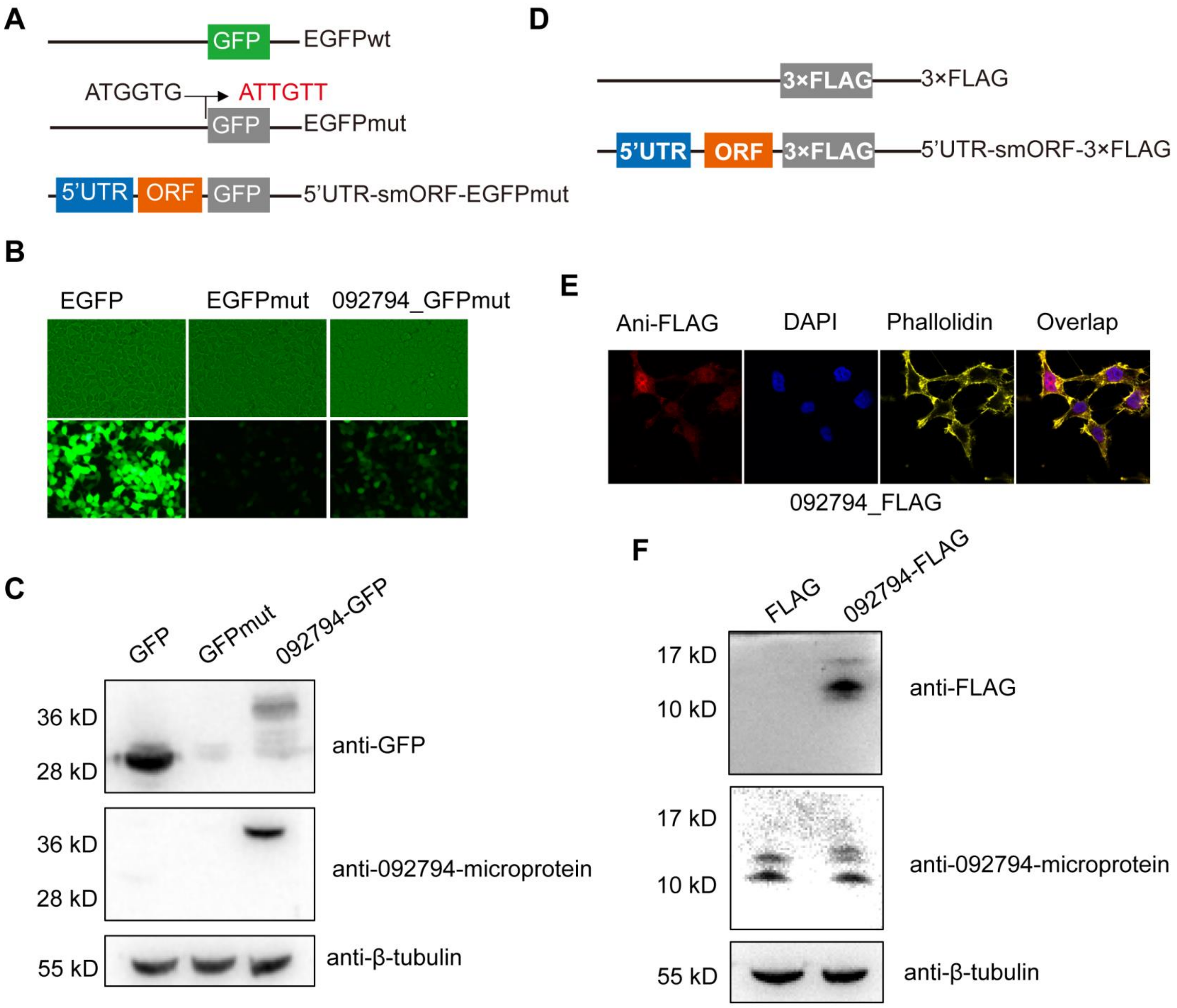

Figure S6. **Experimental validation for the presence of NONHSAT092794-microprotein in 293T cells.** (A) Diagram of the GFP fusion constructs used for transfection. The start codon ATGGTG of the GFP (GFPwt) gene is mutated to ATTGTT (GFPmut). (B) Expression of the NONHSAT092794-GFP fusion protein in NONHSAT092794 5' UTR-smORF-GFPmut-transfected 293T cells. (C) Western blot analysis of cell lysates from 293T cells transfected with different GFP fusion constructs using anti-GFP antibodies, with a 15-µg protein loading volume. β-tubulin was used as a protein loading control. (D) Diagram of the FLAG fusion construct used for transfection. (E) Expression of the NONHSAT092794-FLAG fusion proteins in NONHSAT092794 5' UTR-ORF-FLAG-transfected cells. (F) Western blot analysis of cell lysates from 293T cells transfected with different FLAG fusion constructs using anti-FLAG antibodies, with a 15-µg protein loading volume. β-tubulin was used as a protein loading control.

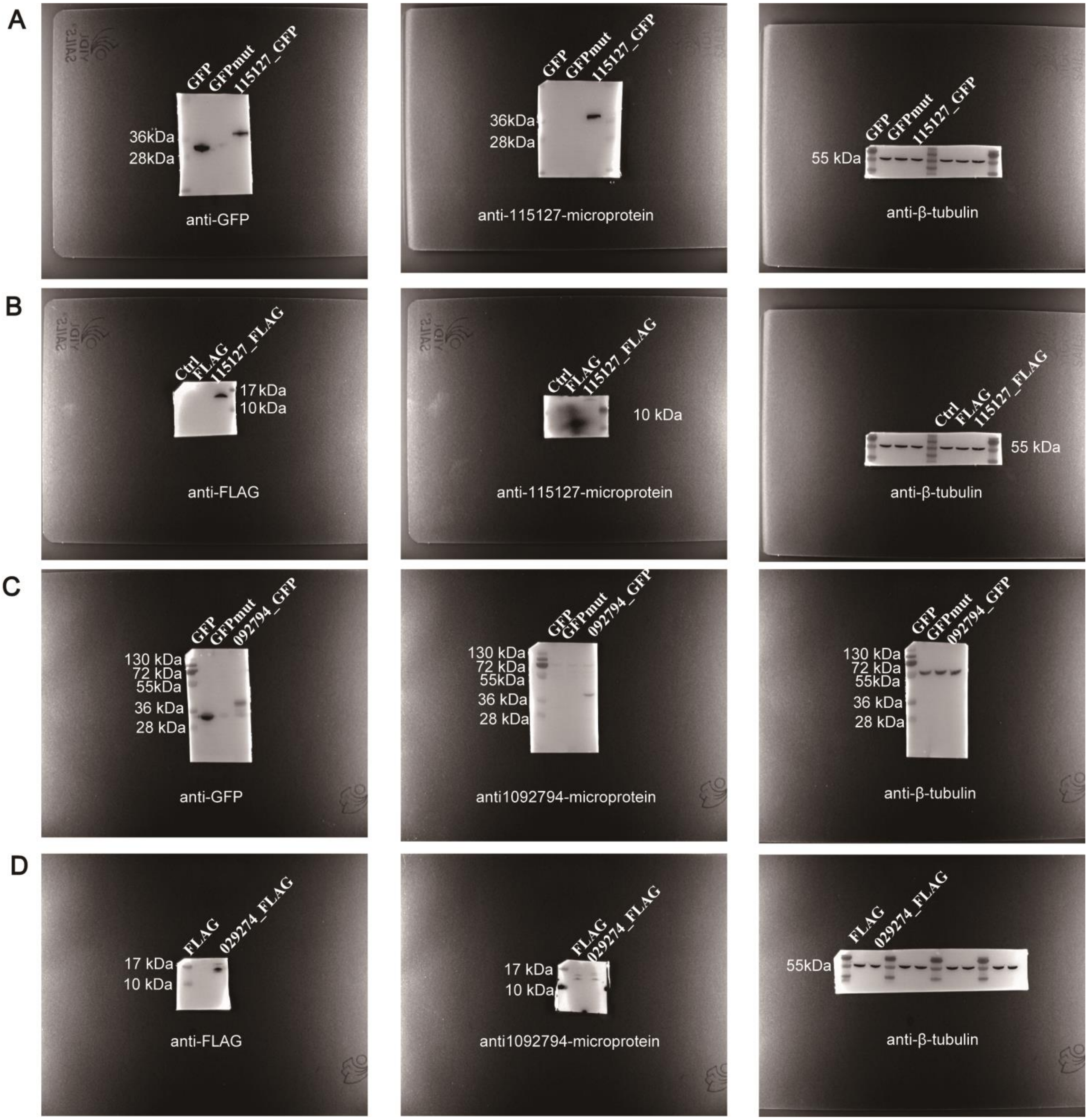

Figure S7. Full size images of Western blot for detection of microprotein-GFP- or -FLAG-fusion proteins. (A) Corresponding to the detection of NONHSAT115127-GFP fusion protein (Figure 4.E). (B) Corresponding to the detection of NONHSAT115127-FLAG fusion protein (Figure 4.H). (C) Corresponding to the detection of 092794-FLAG fusion protein (Supplementary Figure S6.C). (D) Corresponding to the detection of 092794-FLAG fusion protein (Figure S6.FigureS6. F).

> NONHSAT115127-lncRNA-SEP

GSALILHEGEVTVKEDKINALIKAAGVNVEPFWTGLFAKALANVDMGSLICNGGARGPAPA
EGPAPSTNAVPAEEKK

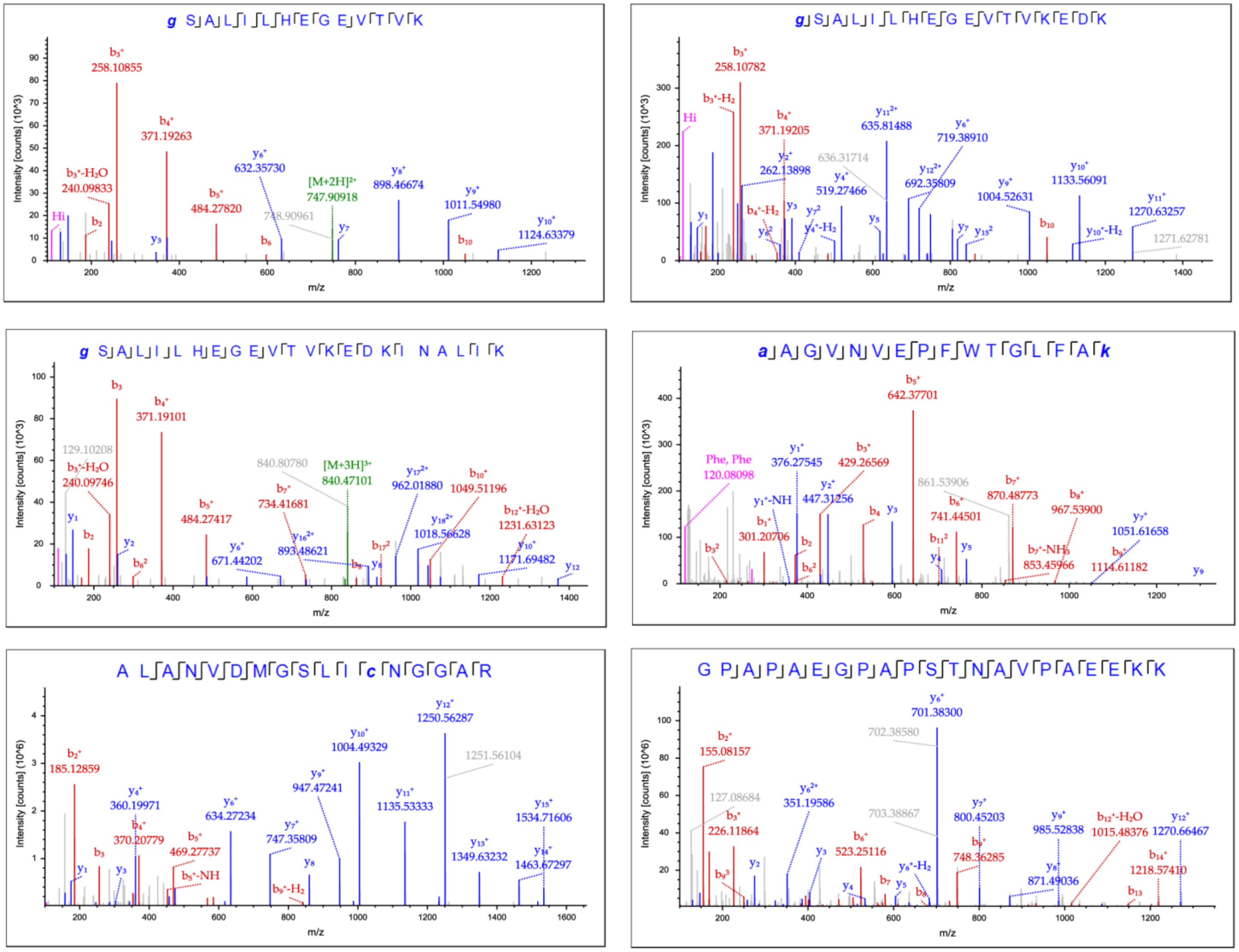

Figure S8. The MS/MS spectra of peptides derived from NONHSAT115127-microprotein. The NONHSAT115127-GFP fusion protein was immunoprecipitated from NONHSAT115127 smORF-GFP-transfected cells by anti-GFP antibody and underwent MS analysis. The results showed that NONHSAT115127-microprotein was identified with 100% sequence coverage.

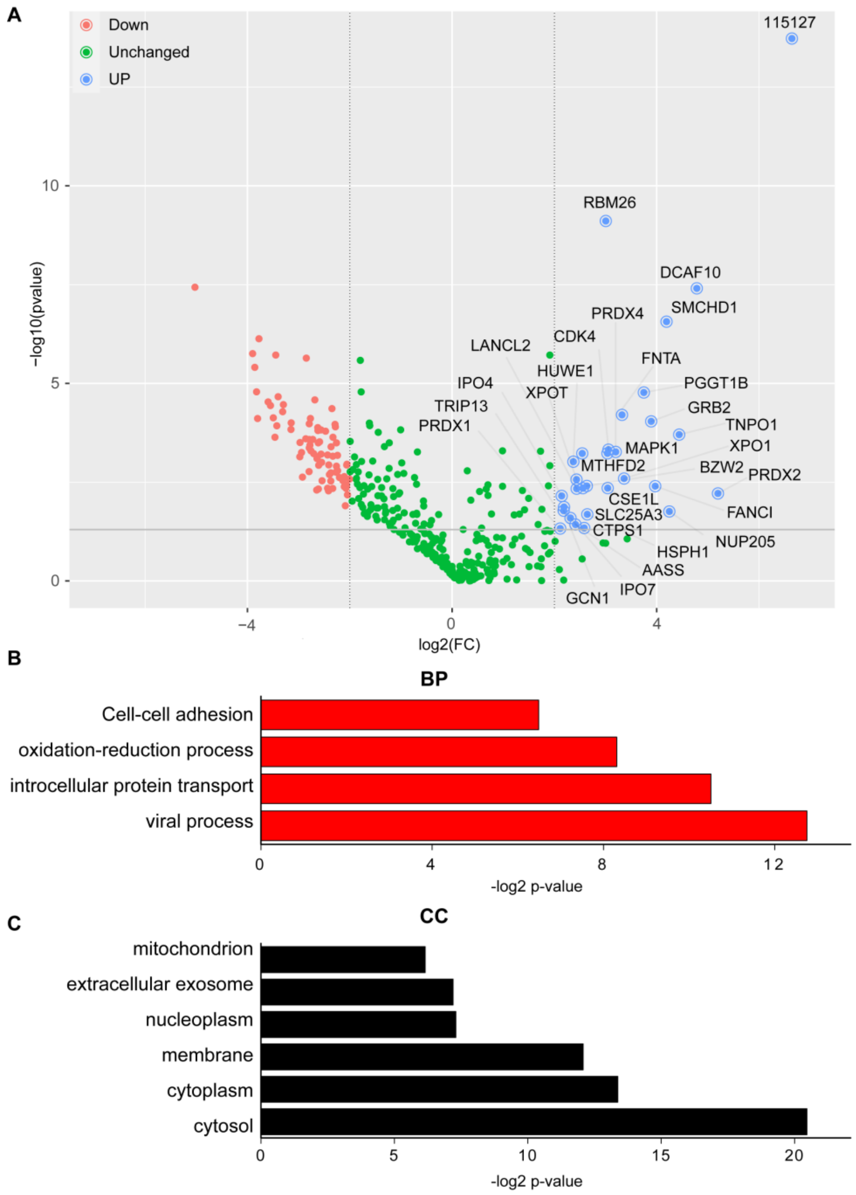

Figure S9. CoIP-MS analysis of proteins potentially interacted with NONHSAT115127-microprotein based on anti-GFP antibodies. (A) Volcano plot comparing the expression levels of proteins that identified in all three bilogical replicates in the co-IP fraction derived from the NONHSAT115127 smORF-GFP- and GFP-transfected cells. Proteins significantly upregulated (Fold change >=3.0, p<= 0.05) in the co-IP fraction derived from the NONHSAT115127 smORF-GFP-transfected cells were shown as blue dots. (B) and (C) GO analysis of the proteins upregulated in the co-IP fraction derived from the NONHSAT115127 smORF-GFP-transfected cells. GO, Gene ontology; BP, biological process; CC: cellular component.

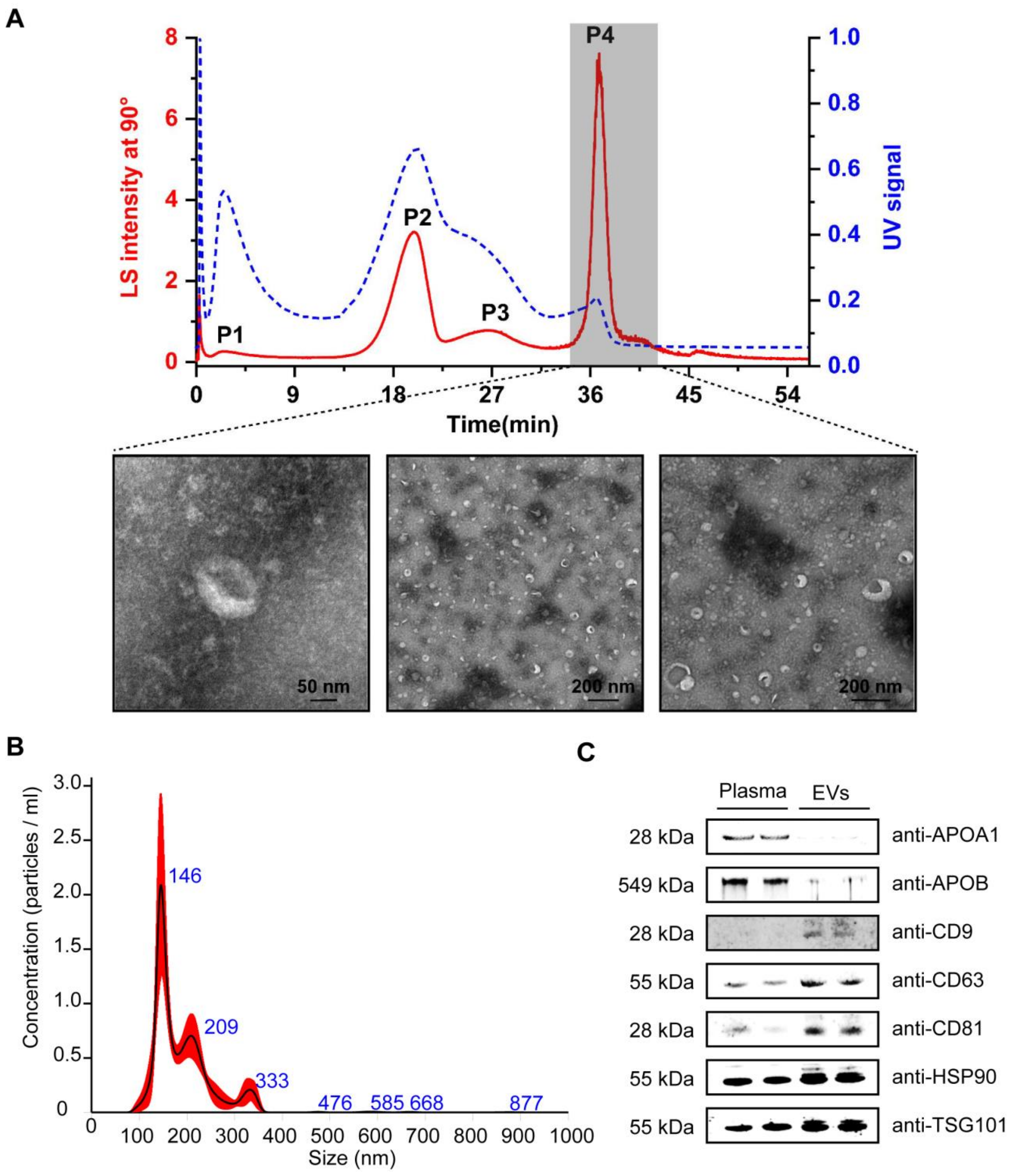

Figure S10. Characterization of plasma EVs isolated by AF4 system. (A) TEM images of plasma EVs (P4 fraction). (B) Nanoparticle tracking analysis (NTA) of plasma EVs. (C) Western blot analysis of P4 fraction and whole plasma with a 15-μg protein loading volume. The results showed enrichment of EV markers (CD9, CD63, CD81, HSP90, and TSG101,) in P4 fraction than whole plasma. Specificity of EVs isolation was confirmed using lipoprotein markers (ApoA1 and ApoB).

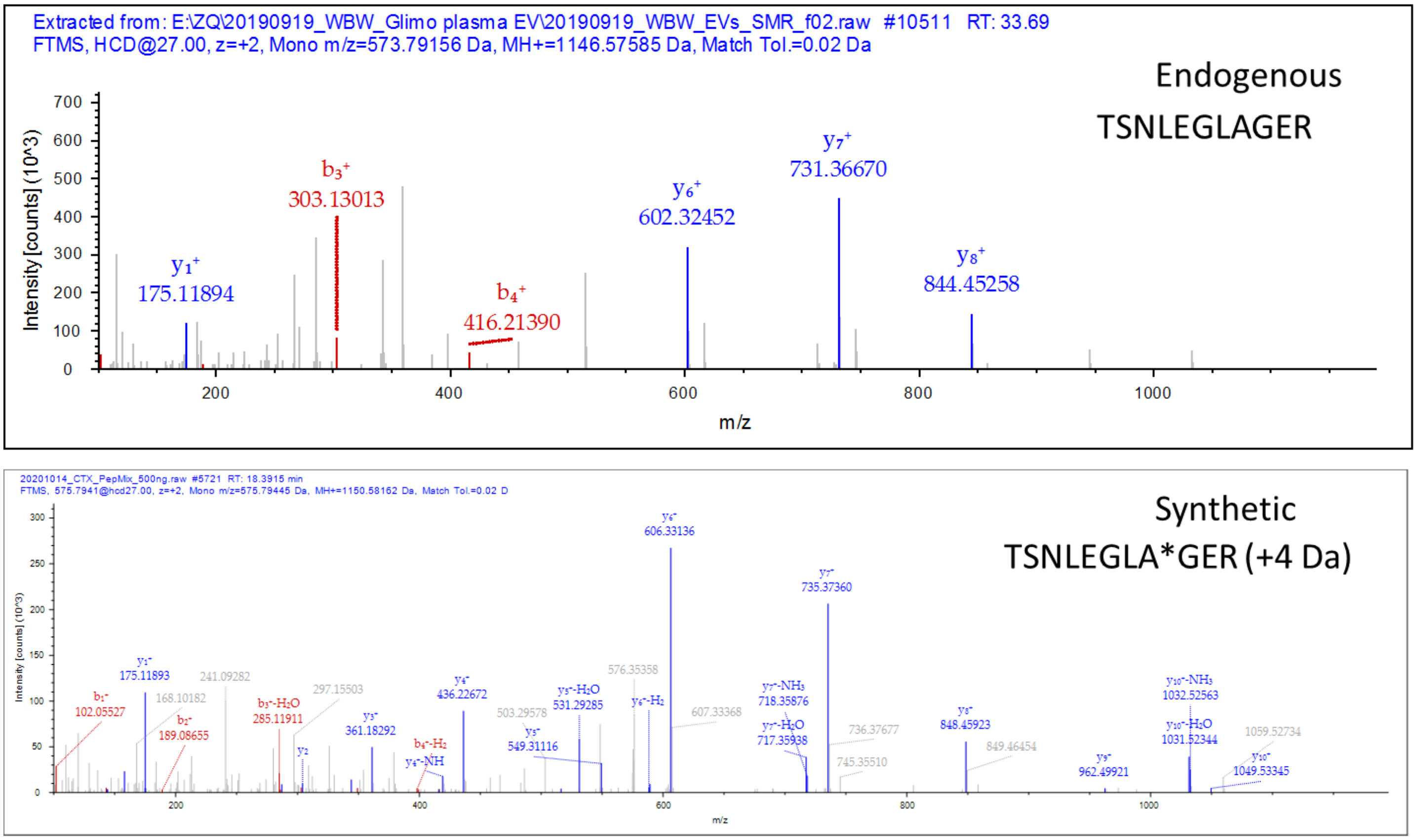

Figure S11. Validation of the identified microproteins in plasma EVs either from healthy donors or glioma cancer patients. The interpreted MS/MS spectra of the endogenous peptide TSNLEGLAGER and the heavy isotope labeled peptide TSNLEGLA*($^{13}C_3{}^{15}N$)GER.

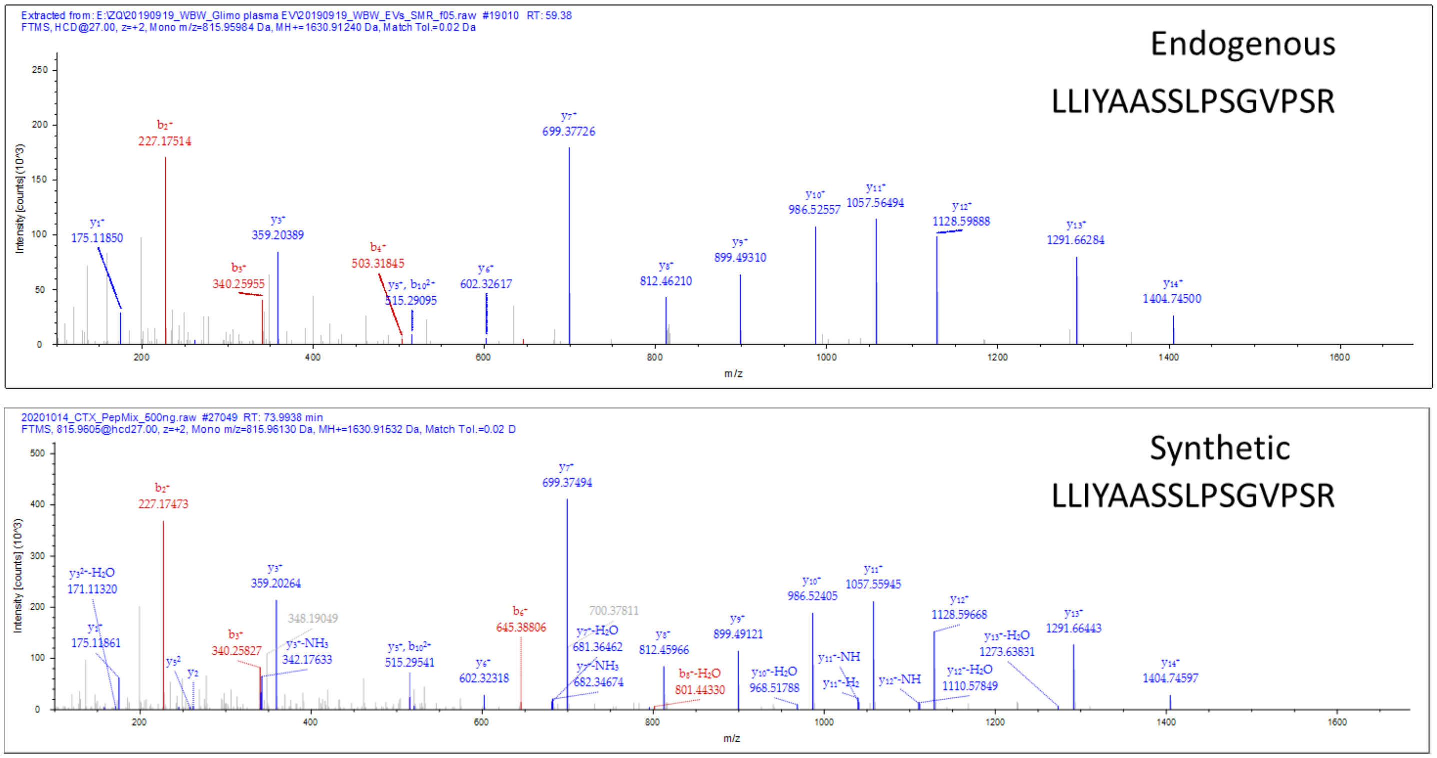

Figure S12. Validation of the identified microproteins in plasma EVs either from healthy donors or glioma cancer patients. The interpreted MS/MS spectra of the endogenous and synthetic peptide of LLIYAASSLPSGVPSR.

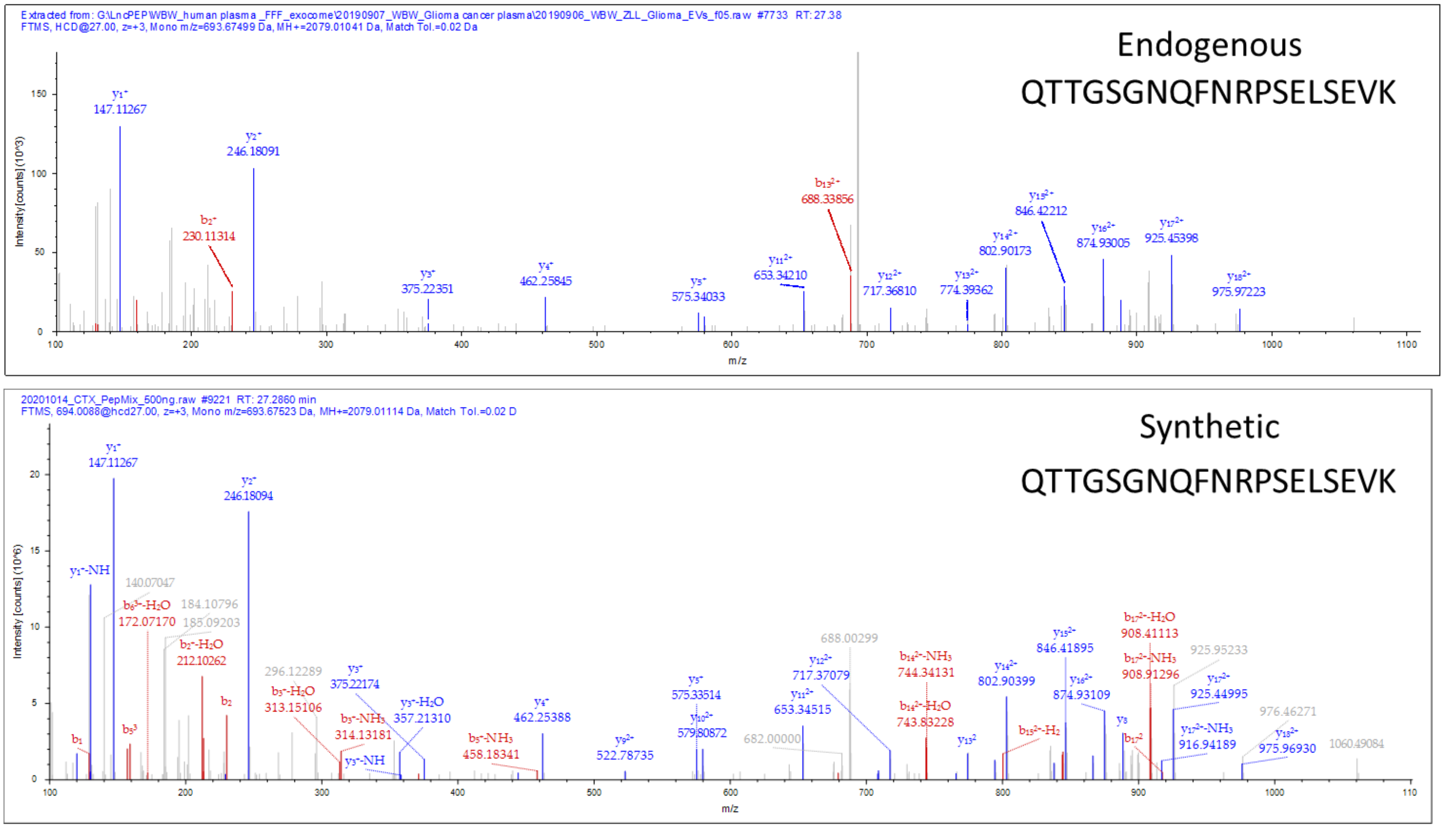

Figure S13. Validation of the identified microproteins in plasma EVs either from healthy donors or glioma cancer patients. The interpreted MS/MS spectra of the endogenous and synthetic peptide of QTTGSGNQFNRPSELSEVK.

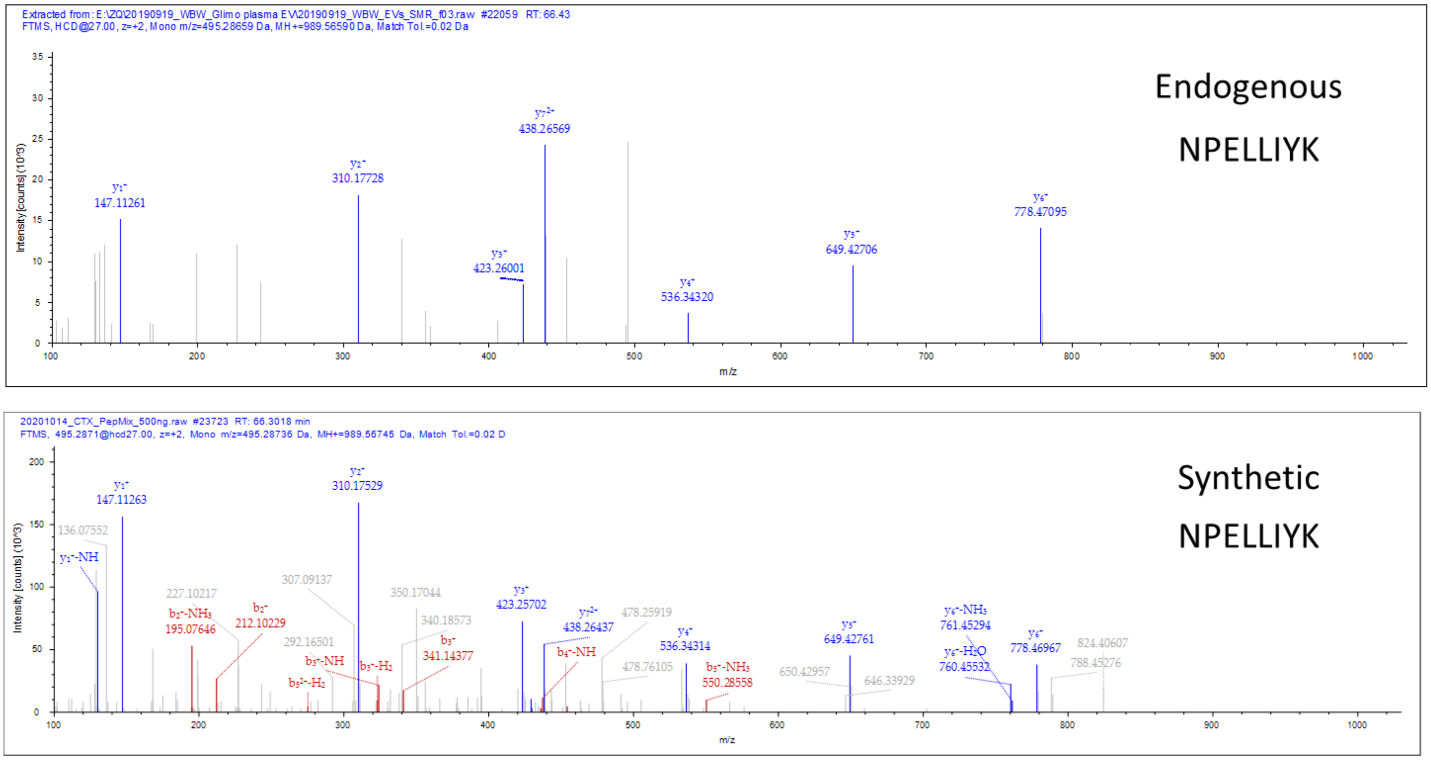

Figure S14. Validation of the identified microproteins in plasma EVs either from healthy donors or glioma cancer patients. The interpreted MS/MS spectra of the endogenous and synthetic peptide of NPELLIYK.

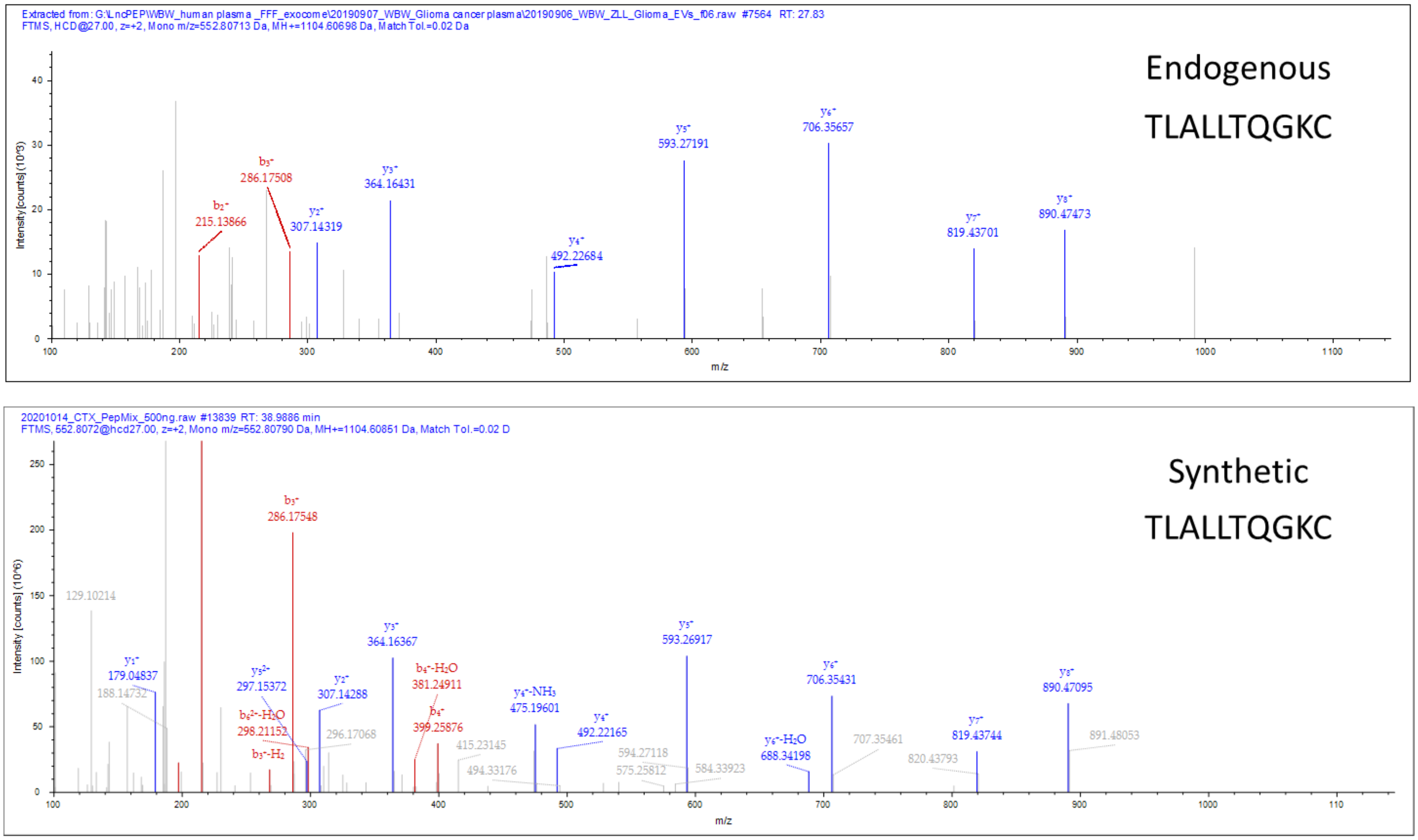

Figure S15. Validation of the identified microproteins in plasma EVs either from healthy donors or glioma cancer patients. The interpreted MS/MS spectra of the endogenous and synthetic peptide of TLALLTQGKC.

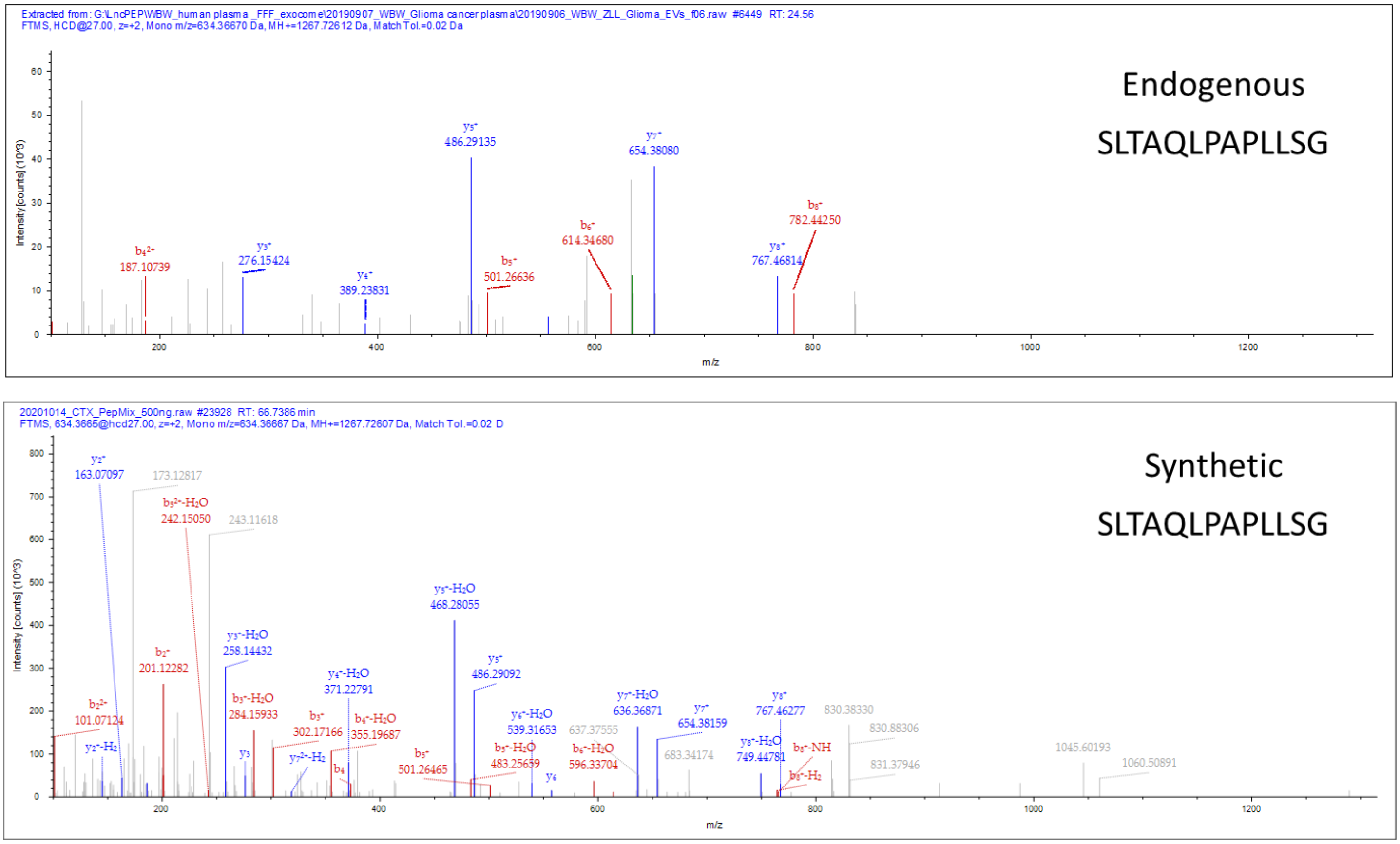

Figure S16. Validation of the identified microproteins in plasma EVs either from healthy donors or glioma cancer patients. The interpreted MS/MS spectra of the endogenous and synthetic peptide of SLTAQLPAPLLSG.

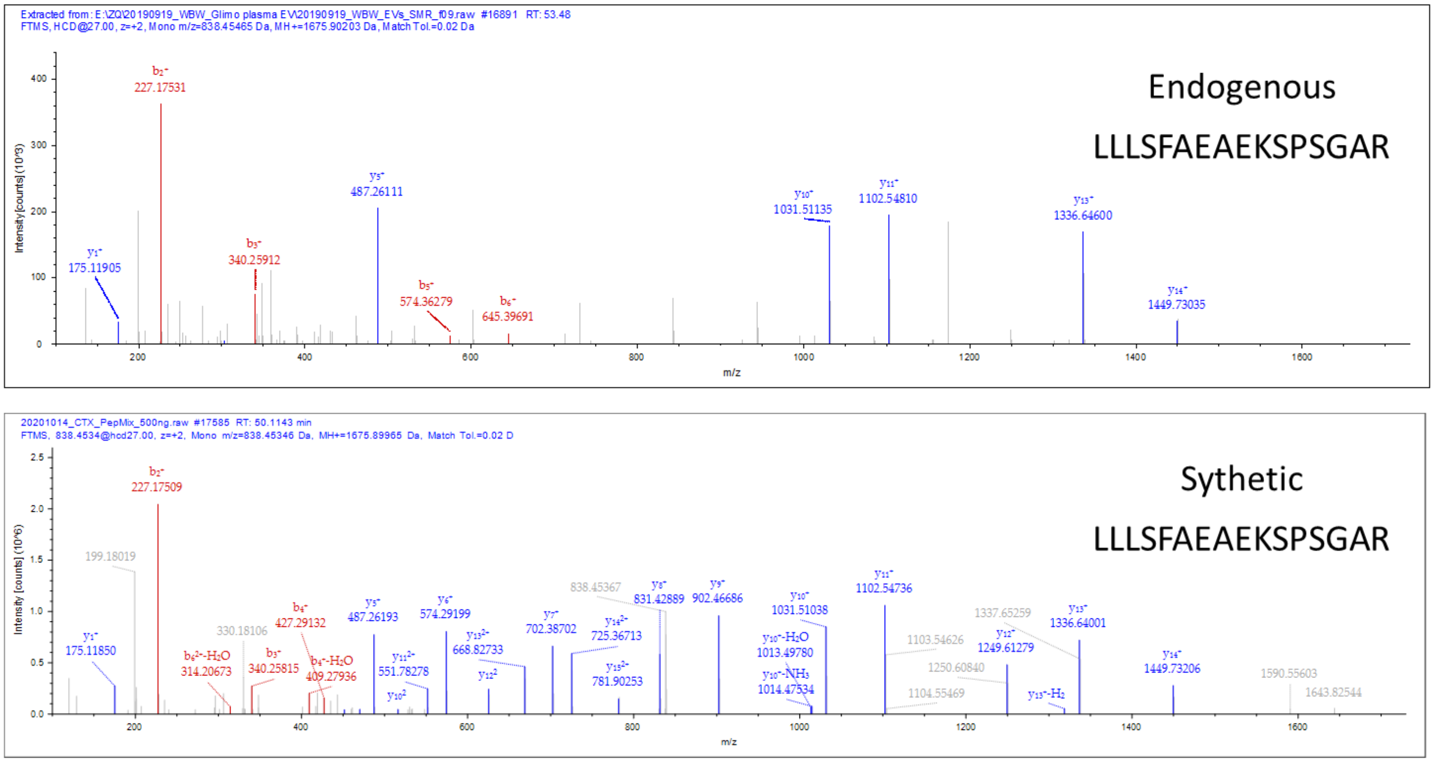

Figure S17. Validation of the identified microproteins in plasma EVs either from healthy donors or glioma cancer patients. The interpreted MS/MS spectra of the endogenous and synthetic peptide of LLLSFAEAEKSPSGAR.

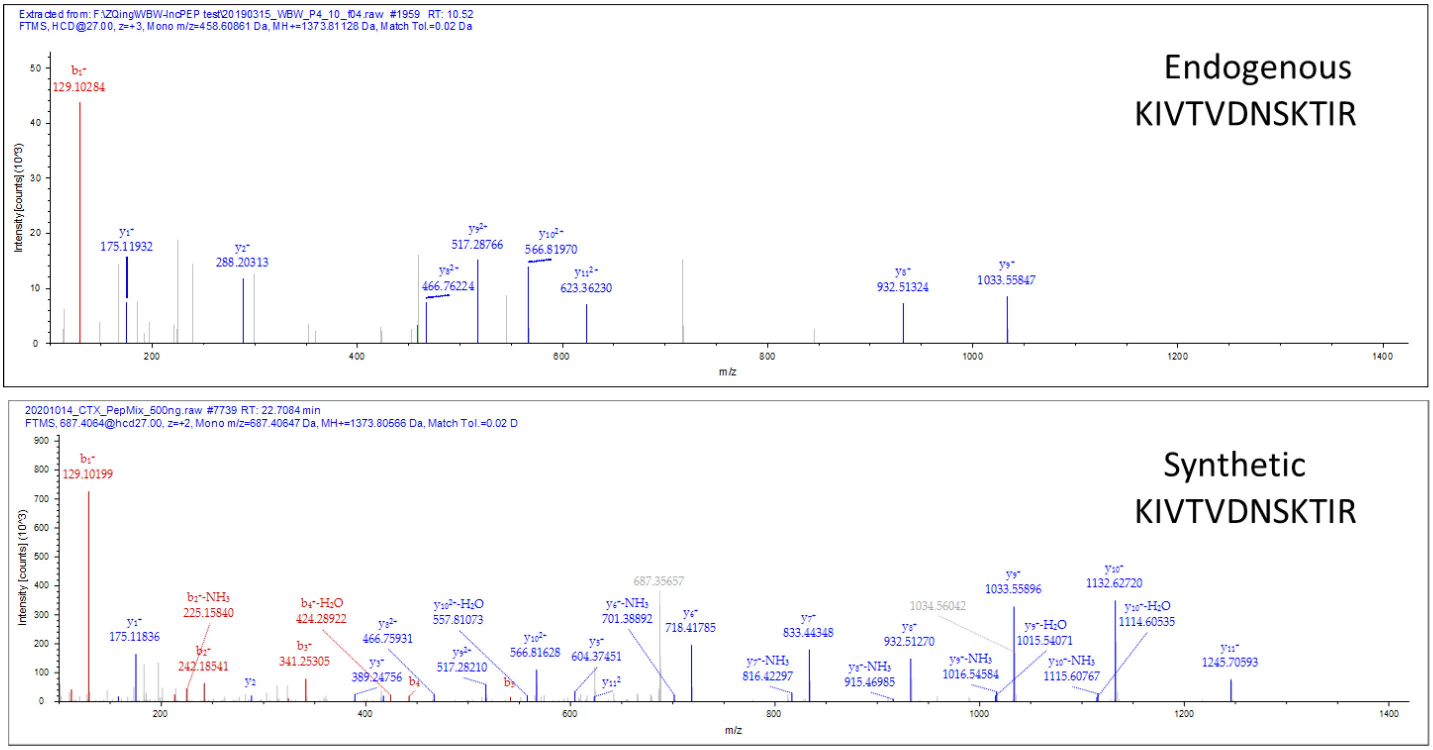

Figure S18. Validation of the identified microproteins in plasma EVs either from healthy donors or glioma cancer patients. The interpreted MS/MS spectra of the endogenous and synthetic peptide of KIVTVDNSKTIR.

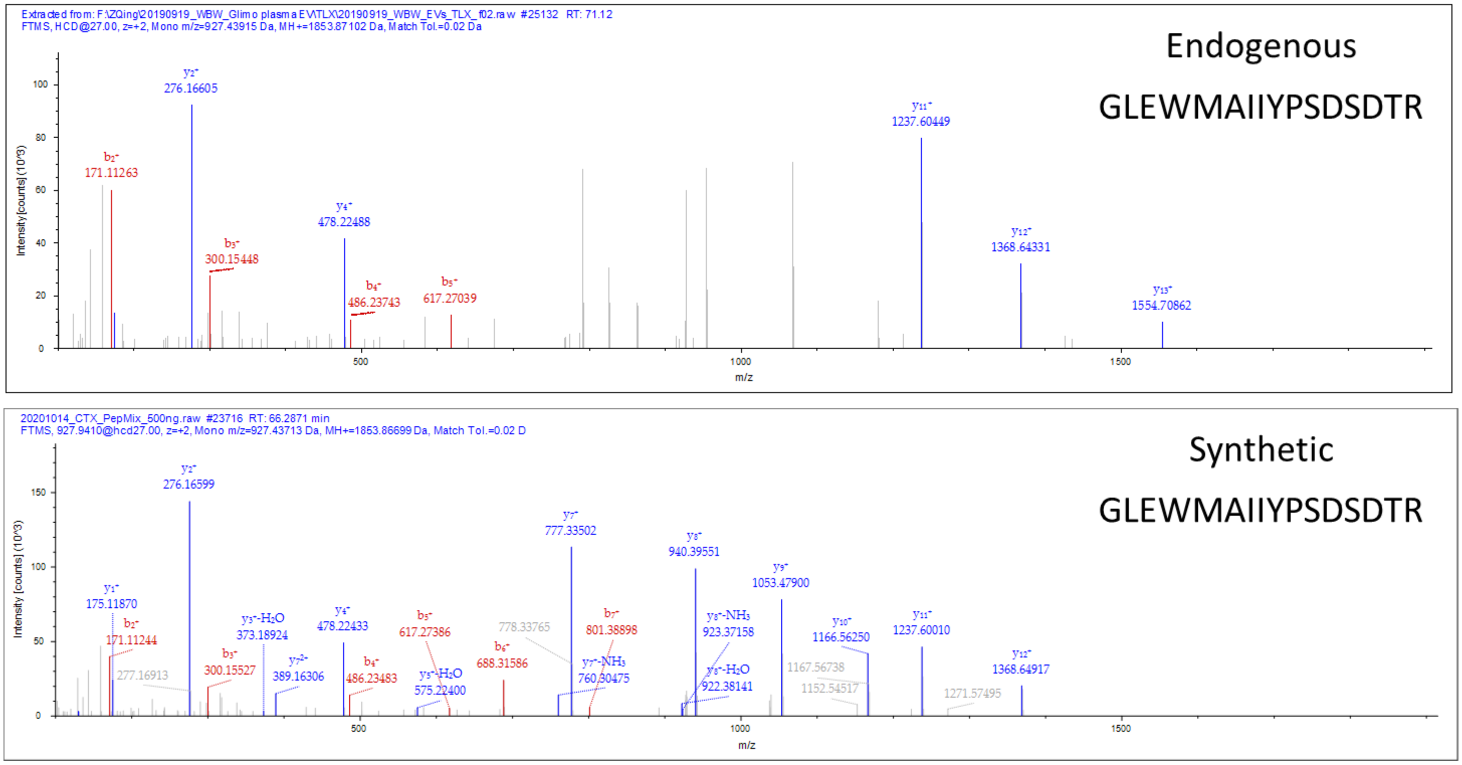

Figure S19. Validation of the identified microproteins in plasma EVs either from healthy donors or glioma cancer patients. The interpreted MS/MS spectra of the endogenous and synthetic peptide of GLEWMAIIYPSDSDTR.

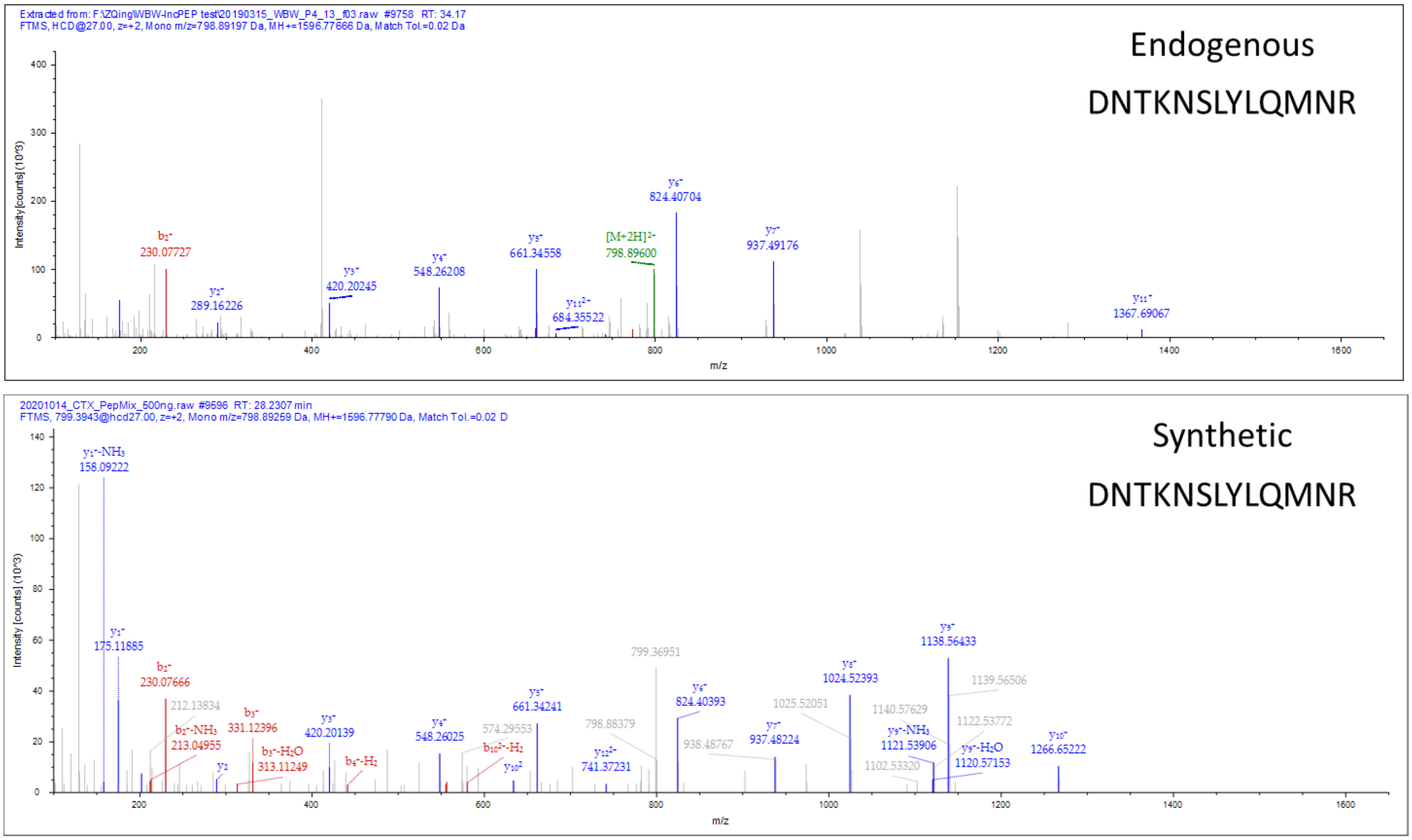

Figure S20. Validation of the identified microproteins in plasma EVs either from healthy donors or glioma cancer patients. The interpreted MS/MS spectra of the endogenous and synthetic peptide of DNTKNSLYLQMNR.

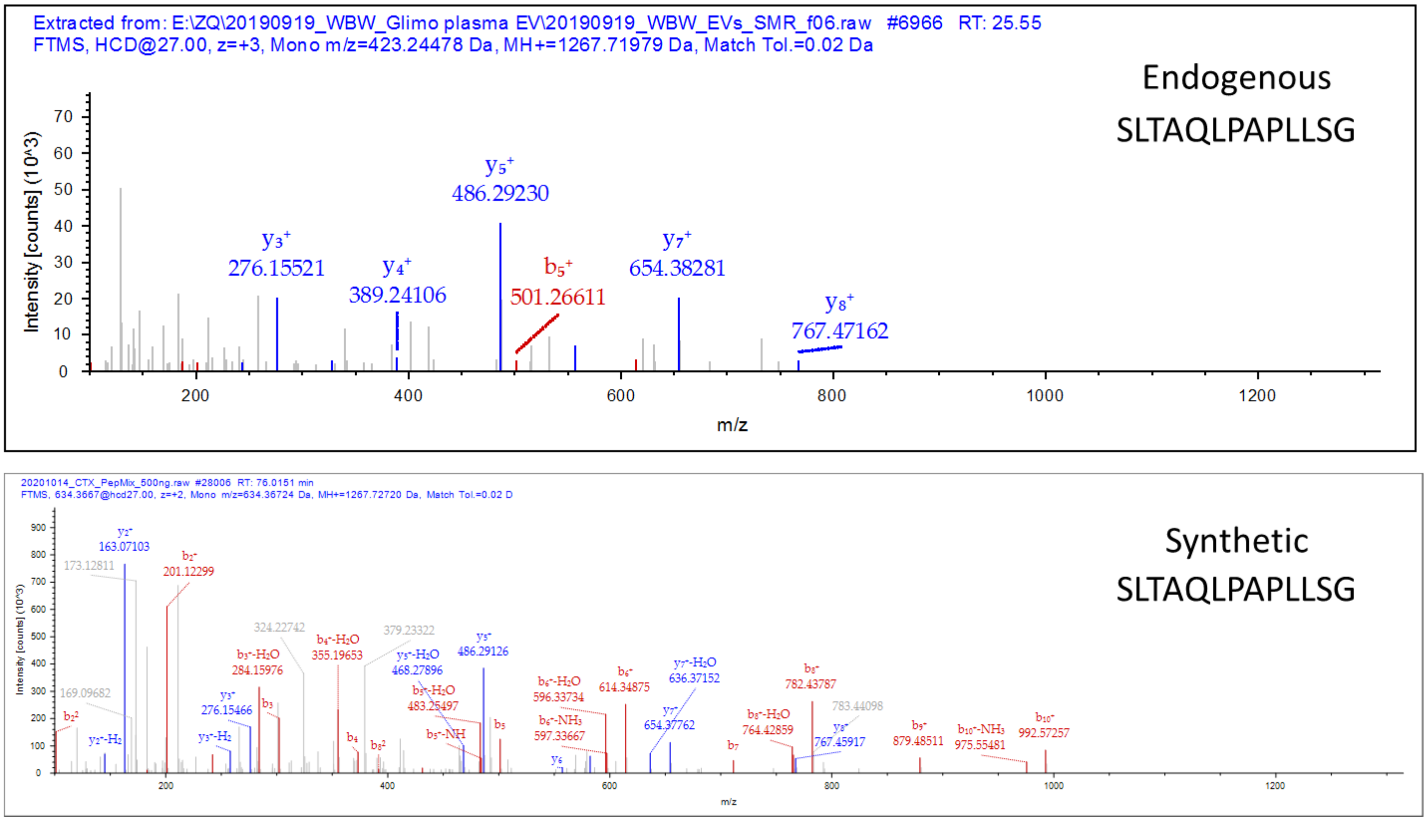

Figure S21. Validation of the identified microproteins in plasma EVs either from healthy donors or glioma cancer patients. The interpreted MS/MS spectra of the endogenous and synthetic peptide of SLTAQLPAPLLSG.

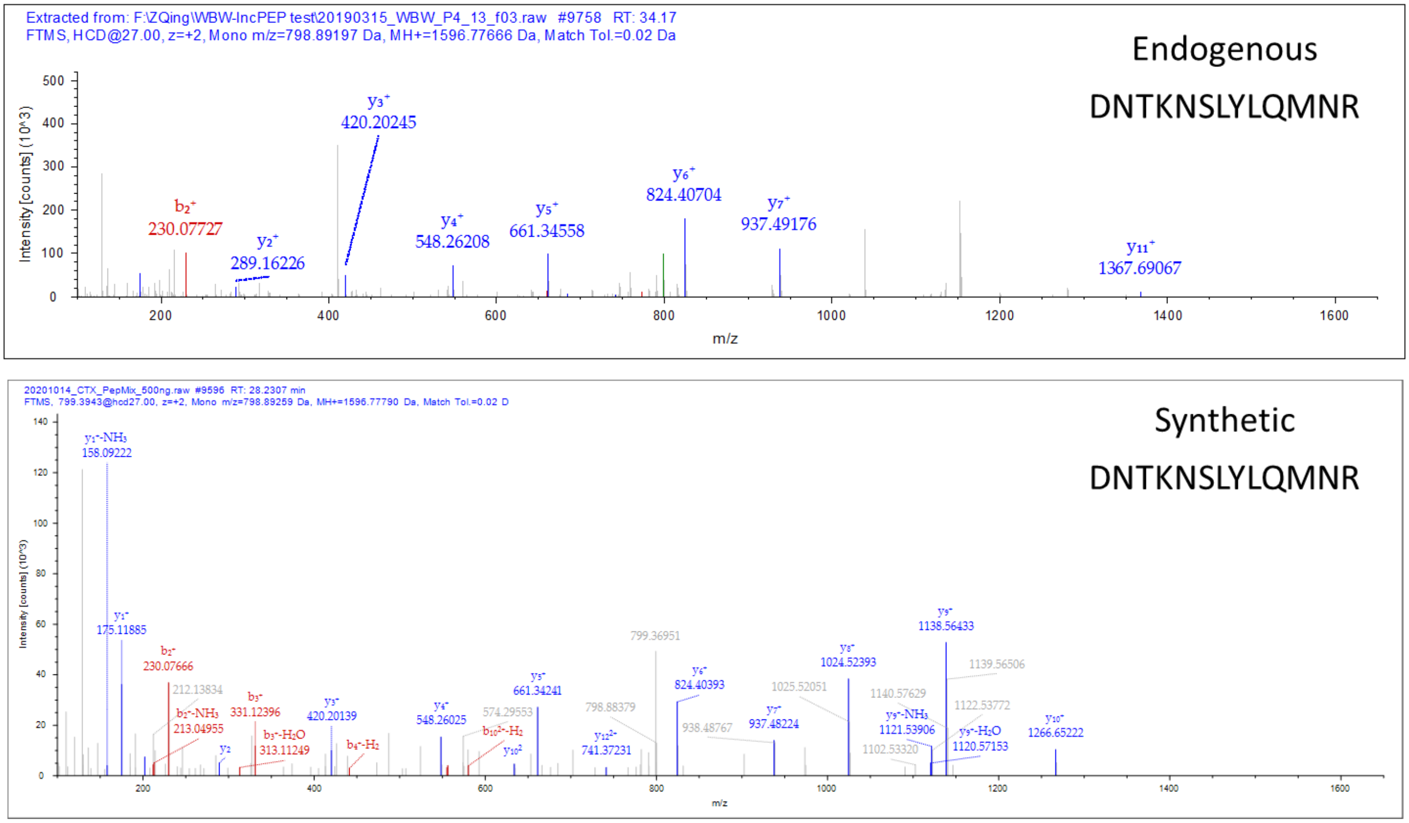

Figure S22. Validation of the identified microproteins in plasma EVs either from healthy donors or glioma cancer patients. The interpreted MS/MS spectra of the endogenous and synthetic peptide of DNTKNSLYLQMNR.
